# Functional humanization of 15-lipoxygenase-1 (Alox15) protects mice from dextran sodium sulfate induced intestinal inflammation

**DOI:** 10.1186/s11658-025-00756-0

**Published:** 2025-07-13

**Authors:** Florian Reisch, Marjann Schäfer, Dominika Labuz, Halina Machelska, Sabine Stehling, Gerhard P. Püschel, Michael Rothe, Dagmar Heydeck, Hartmut Kuhn

**Affiliations:** 1 Department of Biochemistry, Charité – Universitätsmedizin Berlin, corporate member of Freie Universität Berlin and Humboldt Universität Zu Berlin, Charitéplatz 1, 10117 Berlin, Germany; 2 Institute for Nutritional Sciences, University Potsdam, Arthur-Scheunert-Allee 114-116, 14558 Nuthetal, Germany; 3 Department of Experimental Anesthesiology, Charité ˗ Universitätsmedizin Berlin, corporate member of Freie Universität Berlin and Humboldt-Universität Zu Berlin, Hindenburgdamm 30, D-12203 Berlin, Germany; 4 Lipidomix GmbH, Robert-Roessle-Str. 10, 1D-3125 Berlin, Germany

**Keywords:** Eicosanoids, Lipid peroxidation, Oxylipidomes, Inflammation, Pain, Colitis, Paw edema

## Abstract

**Background:**

Mammalian arachidonic acid lipoxygenases (ALOXs) have previously been implicated in the pathogenesis of inflammatory disease, and pro- as well as anti-inflammatory activities have been reported. The human genome involves six functional *ALOX* genes and each of them encodes for a functionally distinct enzyme. ALOX15 is one of these isoforms and the majority of mammalian ALOX15 orthologs including mouse Alox15 convert arachidonic acid to its 12-hydroperoxy derivative. In contrast, human ALOX15 forms 15-hydroperoxy arachidonic acid instead. This difference in the catalytic properties of the two mammalian ALOX15 orthologs may be of biological relevance since arachidonic acid 15-lipoxygenating ALOX-isoforms exhibit an improved biosynthetic capacity for pro-resolving mediators. We recently generated *Alox15* knock-in mice, which homozygously express a humanized Alox15 mutant (Leu353Phe) instead of the wildtype enzyme. These animals should be protected from the development of inflammatory symptoms in whole animal inflammation models if the biosynthesis of pro-resolving mediators plays a major role.

**Methods:**

To explore whether functional humanization of mouse Alox15 might impact the pathogenesis of inflammatory diseases we tested *Alox-KI* mice in comparison with wildtype control animals in two whole animal inflammation models (dextran sodium sulfate induced colitis, Freund’s complete adjuvant induced paw edema). In these experiments we quantified the severity of inflammatory symptoms during the acute phase of inflammation and during the resolution period.

**Results:**

We found that Alox15 knock-in mice are strongly protected from the development of inflammatory symptoms in the dextran sodium sulfate colitis model when the loss of body weight was used as major readout parameter. Quantification of the colon tissue oxylipidomes revealed that the colon concentrations of resolvin D5 were elevated in *Alox15-KI* mice and thus, this mediator might contribute to the protective effect induced by our genetic manipulation. However, other specialized pro-resolving mediators, such as maresin-2, neuroprotectin-1, and lipoxins, may not play a major role for the protective response. In the Freund’s complete adjuvant induced paw edema inflammation model no protective effect was observed.

**Conclusions:**

Taken together, our data suggest that humanization of the reaction specificity of mouse Alox15 (Leu353Phe mutation) exhibits differential effects in two mouse inflammation models.

**Supplementary Information:**

The online version contains supplementary material available at 10.1186/s11658-025-00756-0.

## Background

Arachidonic acid 15-lipoxygenase-1 (Alox15) is one of the seven Alox-isoforms (Alox15b, Alox12, Alox12b, Alox5, Aloxe3, and Aloxe12) for which separate genes exist in the mouse genome [1]. Except for the *Alox5* gene, which is localized on chromosome 6, all other mouse *Alox* genes are constituents of a joint *Alox* gene cluster localized on chromosome 11. Mouse Alox15 was first described some 30 years ago [2] and was originally named leukocyte-type 12-lipoxygenase. Today we know that this enzyme constitutes the mouse ortholog of human ALOX15 [3] and that the two enzymes exhibit inverse reaction specificities when arachidonic acid is used as substrate. Similar functional differences between mouse and human ALOX15 were also observed with other polyenoic fatty acids [4]. The molecular basis for these functional differences has been explored in detail [5–7] and a simple Phe353Leu exchange inverted the reaction specificity of recombinant human ALOX15 favoring AA 12-lipoxygenation [7, 8]. An inverse mutagenesis strategy on recombinant mouse Alox15 (Leu353Phe exchange) humanized the reaction specificity of this enzyme. To explore whether this mutagenesis strategy also humanizes the functionality of mouse Alox15 in vivo we created *Alox15*-knock-in mice (*Alox15-KI*), which express the AA 15-lipoxygenating Leu353Phe mutant of mouse Alox15 instead of the AA 12-lipoxygenating wildtype enzyme [9]**.**

Mammalian ALOX15 orthologs have previously been implicated in the pathogenesis of inflammatory, hyperproliferative and neurological diseases [10–12] and pro- as well as anti-inflammatory functions have been reported [10]. In two different arthritis models systemic functional inactivation of the *Alox15* gene induced uncontrolled inflammation and tissue damage and these data suggested an anti-inflammatory function of Alox15 [13]. Moreover, macrophage specific overexpression of human ALOX15 reduced aortic lipid deposition in a mouse atherosclerosis model, which was paralleled by increased levels of specialized pro-resolving mediators in the atherosclerotic lesions [14]. Virus mediated somatic transfer of the human *ALOX15* gene suppressed the development of inflammatory symptoms and preserved renal function in an experimental model of mouse glomerulonephritis [15]. ALOX15 has also been implicated in the pathogenesis of inflammatory bowel diseases and here anti-inflammatory activities have been reported [16, 17]. Although the mechanistic basis for the observed protective effects is still a matter of discussion the enzyme has been implicated in the biosynthesis of specialized pro-resolving mediators (SPMs), which is consistent with the observed protective activity of the enzyme. On the other hand, *Alox15*^*−/−*^ mice were protected from aortic lipid deposition in two mouse atherosclerosis models [18, 19] and these data suggest a pro-inflammatory function of the enzyme. This conclusion was supported by in vivo atherosclerosis studies, in which human ALOX15 was overexpressed in mouse endothelial cells [20]. Unfortunately, these gain of function studies did not consider the previous observation that mouse and human ALOX15 orthologs exhibit inverse reaction specificities [2, 21] and thus, the observed protective effects might be related to the differential functional characteristics of human and mouse ALOX15.

To explore whether functional humanization of mouse Alox15 may protect mice in different inflammation models we employed our *Alox15-KI* mice [9] and tested these animals in comparison with wildtype control animals in two different in vivo inflammation models. We found that *Alox15-KI* mice developed less intense inflammatory symptoms in the dextran-sulfate-sodium (DSS) induced colitis model and that this protective effect was paralleled by higher colon tissue concentrations of the specialized pro-resolving mediator (SPM) resolvin D5 in the *Alox15-KI* mice. However, other SPMs, such as maresin-2 (Mar-2) and neuroprotection-1 (NPD-1), were not elevated in these animals. In the Freund’s complete adjuvant (CFA) induced paw edema model we did not observe significant differences in the severity of inflammatory symptoms when the two genotypes were compared.

## Methods

### Chemicals and devices

The chemicals and devices used for the experiments were obtained from the following vendors: PBS from PAN Biotech (Aidenbach, Germany); HPLC solvents from Fisher Scientific GmbH (Schwerte, Germany); HPLC standards of 15R/S-HETE, 12R/S-HETE, 8-R/S-HETE and arachidonic acid (AA) from Cayman Chemicals (distributed by Biomol GmbH, Hamburg, Germany); dextrane sulfate sodium (DSS, molecular weight = 36.000–50.000) from ICN Biomedicals (Irvine, USA); Freund’s complete adjuvant (desiccated *M. butyricum*) from Fisher Scientific GmbH (Schwerte, Germany); Nucleospin RNA plus kit (Macherey–Nagel, Düren, Germany); reverse transcriptase kit (Meridian Bioscience, Memphis, USA); the plethysmometer model 37140 from Ugo Basile (Gemonio, Italy); von Frey cages (Model 410) and Hargreaves cages (Model 336) from IITC Life Sciences (Woodland Hills, Los Angeles, USA).

### Animals

The Crispr/Cas9 strategy was used to mutate the *Alox15* gene in vivo so that the resulting knock-in mice express the AA 15-lipoxygenating Leu353Phe Alox15 mutant. A more detailed description of the mutagenesis strategy and basic characterization of the knock-in animals (*Alox15-KI* mice) are given in [9]. In brief, for production of the *Alox15-KI* mice the Cas9 mRNA, the gRNA generated by in vitro transcription and the donor oligonucleotide were co-injected into fertilized eggs of C57BL/6 J-mice. Heterozygous allele carriers were selected and were inter-crossed to obtain homozygous *Alox15-KI* mice and homozygous wildtype control animals. These homozygous founder animals were separately intercrossed and a colony of *Alox15-KI* mice as well as a colony of wildtype controls were established. Both colonies were on C57BL/6 J background and the mice were kept under identical experimental conditions next to each other in the same room of the animal house of Charité. Each individual mouse included in the experiments was separately genotyped.

### Recombinant expression of wildtype and mutant (Leu353Phe) Alox15 variants and in vitro activity assay

Wildtype mouse Alox15 and its AA 15-lipoxygenating Leu353Phe mutant were expressed in *Escherichia coli* as N-terminal his-tag fusion proteins. For this purpose, the coding region of the mouse *Alox15* cDNA was cloned into the bacterial expression plasmid pET28b and competent bacteria [Rosetta 2 DE3 pLysS] were transformed with the recombinant plasmid. A well-separated bacterial clone was selected and a 1 ml bacterial pre-culture (LB medium with 50 μg/ml kanamycin and 35 μg/ml chloramphenicol) was grown at 37 °C for 6 h at 180 rpm agitation. After the pre-culture reached an optical density (OD600) of 5, the pre-culture was added to a 50 ml main culture, which was grown overnight at 30 °C in Ultra Yield flasks (Thomson Instrument Company, Oceanside, USA). Expression of the recombinant enzymes was induced by the addition of 1 mM (final concentration) isopropyl-*β*-D-thiogalactopyranosid (IPTG). Bacteria were harvested by centrifugation, the resulting pellet was reconstituted in 5 ml PBS, cells were lysed by sonication and aliquots of the lysis supernatant were used for in vitro activity assays.

To humanize the reaction specificity of recombinant mouse Alox15 a Leu353Phe exchange was carried out using the PfuUltra II Hotstart PCR Master Mix kit (Agilent Technologies Germany GmbH & Co. KG, Waldbronn, Germany) and the following mutagenesis primers were designed: forward, 5′-GTC CGA AGC TCA GAC TTC CAG CTT CAT GAG-3′; reversed, 3′- CTC ATG AAG CTG GAA GTC TGA GCT TCG GAC -5′. Mutagenesis PCR was performed as follows: 95 °C for 2 min initial denaturation, cycle: 30 s at 95 °C (denaturation phase), then 60 s at 55 °C (annealing phase) followed by the synthesis phase (10 min at 68 °C). This cycle was repeated 16 times. Subsequently, the amplification products were digested with 1 μL DpnI (Thermo Scientific, Schwerte, Germany) for 30 min at 37 °C. Then, 8 μL of the PCR sample were used for transformation of competent *E. coli* XL-1 Blue cells (Agilent Technologies Inc., Santa Clara, USA). The mutant plasmid was isolated and after sequencing the Leu353Phe mutant Alox15 was expressed.

For in vitro activity assays aliquots of the bacterial lysate supernatants of wildtype Alox15 and its Leu353Phe mutant were incubated in 0.5 ml PBS containing 100 µM arachidonic acid as substrate. After 3 min of incubation the hydroperoxy lipids formed during the Alox-reaction were reduced to the corresponding hydroxy compound by the addition of 1 mg solid sodium borohydride. After acidification with 35 µl of acetic acid protein was precipitated by the addition of 0.5 ml of acetonitrile and aliquots of the protein-free supernatant were injected to RP-HPLC analysis.

### Ex vivo* Alox15* activity assays

In mice Alox15 is high level expressed in peritoneal lavage cells and in bone marrow cells [22]. Thus, we carried out ex vivo activity assays using these cells prepared from *Alox15-KI* mice and of wildtype control animals as enzyme source. To obtain peritoneal lavage cells we sacrificed four *Alox15-KI* mice and four wildtype controls under anesthesia, injected 10 ml of PBS into the peritoneal cavity and gently massaged the belly for about 2 min. Then, the cell suspension was aspirated (typically 8–9 ml were recovered), cells were spun down and washed twice with PBS. Finally, the cell pellet was dissolved in 1 ml of PBS containing 100 µM of AA and incubated for 15 min at room temperature. The reaction was stopped with 1 mg of solid sodium borohydride, the sample was acidified (35 µl acetic acid) and protein was precipitated by the addition of 1 ml acetonitrile. Precipitate was spun down and aliquots of the protein-free supernatants were injected to RP-HPLC.

To obtain bone marrow cells the two femur bones were freed from adhering connective tissue and the two condyles were cut off. The bone marrow cavity was rinsed with 10 ml PBS each, the cells were pelleted, washed twice with PBS and reconstituted in 0.5 ml PBS. The cells prepared from the two femur bones were combined, the cells were spun down, the cell pellet was resuspended in 1 ml PBS containing 100 µM AA and the suspensions were incubated for 15 min at room temperature. The reaction was stopped with 1 mg of solid sodium borohydride, the sample was acidified (35 µl acetic acid) and protein was precipitated by the addition of 1 ml acetonitrile. Precipitate was spun down and aliquots of the protein-free supernatants were injected to RP-HPLC.

RP-HPLC was carried out using a Shimadzu instrument (LC20 AD) that was equipped with a diode array detector (SPD M20A). The hydroxy fatty acids were separated on a Nucleodur C18 Gravity column (Macherey–Nagel, Düren, Germany; 250 × 4 mm, 5 μm particle size) coupled with a guard column (8 × 4 mm, 5 μm particle size). A solvent system consisting of acetonitrile: water: acetic acid (70:30:0.1, by vol) was employed at a flow rate of 1 ml/min and analytes were eluted isocratically at 25 °C. The conjugated dienes formed during the incubation period were prepared, the solvents were removed and the remaining lipids were reconstituted in 200 µl of hexane containing 0.1% acetic acid. To resolve the hydroxy fatty acid isomers formed during the incubation period combined normal phase/chiral phase HPLC (NP/CP-HPLC) was carried out. For this purpose, a Chiralpak AD-H column (4.6 × 250 mm, 5 µm particle size, Daicel (Osaka, Japan) was connected with a Nucleosil pre-column (4.6 × 30 mm, 5 µm particle size, Macherey–Nagel (Düren, Germany) and the analytes were eluted isocratically using a solvent system consisting of n-hexane/methanol/ethanol/acetic acid (96/3/1/0.1, by vol) at a flow rate of 1 ml/min. The absorbance at 235 nm was monitored and the retention times of authentic standards are indicated above the chromatographic traces.

### RNA extraction and qRT-PCR

10–15 mg (wet weight) of inflamed colon and paw tissue were prepared at different time points of the inflammatory reaction and stored in RNAlater solution (Sigma-Aldrich/Merck, Taufkirchen, Germany) at − 20 °C. After thawing the tissue was cut into small pieces using a scalpel and was then homogenized in 400 µl LBP buffer (Nucleospin RNA plus kit, Macherey–Nagel, Düren, Germany) using a FastPrep24 homogenizer. Cell debris was spun down and from the homogenate supernatant total RNA was extracted following the instructions of the vendor of the Nucleospin RNA plus kit (Macherey–Nagel, Düren, Germany). Subsequently, 500 ng RNA were reversely transcribed using the Tetro Reverse Transcriptase kit (Meridian Bioscience, Memphis, USA, distributed by BioCat GmbH, Heidelberg, Germany) and Oligo dT_18_ reagents as recommended by the vendor. qRT-PCR was performed using intron-spanning amplification primer combinations for the different target genes, which were synthesized by BioTez GmbH, Berlin, Germany. External amplification standards were cloned for each target gene and known amounts of these standards were added to the amplification mixture. The following primer combinations were used for quantification of the different pro-inflammatory gene products: iNOS: 5′-TGA GGC CCA GGA GGA GAG AGA-3ʺ and 3′-TCA CAG GCT GCC CGG AAG GTT-5′; TNFalpha: 5′-CAC CAT GAG CAC TGA AAG CAT GAT-3′ and 3′-CGG CTG ATG GTG TGG GTG AGG-5; IL-1beta: 5′-CTA CAG GCT CCG AGA TGA ACA ACA AAA-3′ and 3′-TGG GGA ACT CTG CAG ACT CAA ACT C-5; IL-6: 5′-AAC CAC GGC CTT CCC TAC TTC A-3′ and 3′-TCT GGC TTT GTC TTT CTT GTT ATC T-5. The concentrations of target cDNAs were quantified using standard curves (known copy numbers of the external amplification standards) and the amounts of amplification products were normalized to Gapdh expression. qRT-PCR was performed on a Rotor Gene 3000 device (Corbett Research, Mortlake, Australia). The progress of the amplification process was followed online using the SensiMix^™^ SYBR PCR Kit (Meridian Bioscience, Memphis, TN, USA, distributed by BioCat GmbH, Heidelberg, Germany).

### Dextrane-sulfate sodium (DSS) induced experimental colitis

The experiment was approved by the responsible State animal care committee (Landesamt für Gesundheit und Soziales, Berlin, Germany) and registered under permission number G 0272/19. In the DSS induced colitis model [23, 24] intestinal inflammation is induced by repeated oral application of DSS in the drinking water and different clinical readout parameters can be quantified to judge the severity of the inflammatory reaction. Since the degree of colitis depends on the purity of the commercial DSS, on the functional status of the mice and on the DSS concentration in the drinking water we first performed preliminary experiments (three female wildtype mice) testing the appropriate DSS concentration. In these experiments we found that 1.5% DSS in the drinking water induced subtle but clearly measurable inflammatory symptoms. When we used 2% DSS in the drinking water strong inflammatory symptoms were observed and thus, in agreement with the animal welfare regulations, we decided to perform the main experiments using 1.5% DSS in the drinking water as inflammatory stimulus.

To perform the main experiment, we randomly selected 12 female individuals (age of 12–15 weeks) from both, our colony of *Alox15-KI* mice and of the colony of wildtype control animals. We set up two cages for both genotypes and each cage involved the animals of one experimental group. Cage A: six wildtype mice representing the acute inflammation phase; cage B: six wildtype mice representing the inflammatory resolution phase; cage C: six *Alox15-KI* mice representing the acute inflammation phase; and cage D: six *Alox15-KI* mice representing the inflammatory resolution phase. In addition, two 0 day control groups (*Alox15-KI* 0 day control group, wildtype 0 day control group) each with six individuals were set up. At the beginning of the experiments (day 0 of the experimental protocol) the body weights of all animals were determined and since there was no significant difference between the body weights of *Alox15-KI* and wildtype mice the mean of all body weights was set 100%. After weighing the mice of the two 0 day control groups were sacrificed under anesthesia and the other mice received 1.5% DSS in the drinking water for 8 consecutive days. To profile the body weight kinetics the animals were weighted each day in the morning and their health status (mobility) was monitored. After 8 days of DSS treatment the animals of cages A (wildtype acute inflammation phase) and C (*Alox15-KI* acute inflammation phase) were sacrificed under anesthesia, the colons were prepared and washed with PBS. For cages B and D representing the resolution phase of wildtype control mice and *Alox15-KI* mice, respectively, the DSS containing drinking water was exchanged by normal drinking water and the animals were allowed to recover from intestinal inflammation.

After preparation the colons were washed twice with PBS and were divided into four segments of similar length. The distal segments were each transferred to 0.5 ml of an RNAlater solution and were incubated for 30 min at room temperature. Then the samples were shock-frozen in liquid nitrogen for future preparation of total RNA and for subsequent gene expression studies. The two middle colon sections were immediately shock-frozen in liquid nitrogen for later oxylipidome profiling and for tissue protein extraction (immunoblotting of relevant gene products). The proximal segment was transferred to 1 ml of a 4% paraformaldehyde solution for preparation of histological cross sections.

### Solid phase tissue lipid extraction

After thawing about 10 mg of colonic tissue was added to 490 µl of water [25, 26]. Then 10 µl of an internal standard solution (LTB4-d4, 20-HETE-d6, 15-HETE-d8, 13-HODE-d4, 14,15-DHET-d11, 9,10-DiHOME-d4, 12,13-EpOME-d4, 8,9-EET-d11, PGE2-d4; 10 ng/ml each) and 5 µl of a methanolic solution of tert-butyl hydroxy toluene (1 mg/ml) were added. Afterwards 300 µl of water, 100 µl of 10 M NaOH solution and 500 µl methanol were added. The sample was incubated for 30 min at 60 °C (alkaline hydrolysis of the cellular ester lipids), put on ice for 5 min and acidified with 100 µl of 58% acetic acid. Afterwards, 2 ml of 0.1 M phosphate buffer was added and the pH was adjusted to 6.0 by addition of small aliquots of acetic acid or NaOH. A 50 µl aliquot was removed for later determination of the protein concentration. The rest of the sample was centrifuged and the protein free supernatant was used for solid phase lipid extraction on a combined reverse phase-ion exchange Agilent Bond-Elut-Certify II cartridge (200 mg, Agilent Technologies, Santa Clara, CA, USA). Before sample application the cartridge was first rinsed with 3 ml of methanol and then conditioned with 3 ml 0.15 M phosphate buffer, pH 6.0. After sample application the cartridge was rinsed with 3 ml of a 1:1 mixture of water and methanol. Elution of the tissue lipids was carried out by rinsing the cartridge with a 74: 25: 1 (by vol) mixture of ethyl acetate: n-hexane: acetic acid. The solvents were evaporated under a stream of nitrogen and the remaining lipids were reconstituted in 100 µl of a 3: 2 mixture (by vol) of methanol and water.

### LC–MS/MS based oxylipidome analyses

To explore the patterns of the colon oxylipins we quantified the amounts of more than 40 different oxygenated PUFA derivatives [25, 26]. Liquid chromatography–tandem mass spectrometry (LC–MS/MS) was performed on an Agilent 1290/II LC–MS system consisting of a binary pump, an autosampler and a column oven (Agilent Technologies, Waldbronn, Germany). For chromatographic separation, we employed an Agilent Zorbax Eclipse C_18_ UPLC column (150 × 2.1 mm, 1.8 µm particle size). The analytes were eluted at 30 °C with a solvent system that was mixed from two solvent stock solutions. Stock A: water containing 0.05% acetic acid. Stock B: 1:1 mixture (by vol.) of methanol: acetonitrile. The HPLC system was connected with a triple quadrupole MS system (Agilent 6495 System, Agilent Technologies, Santa Clara, USA) that was run in the negative electrospray ionization mode. Each metabolite was detected simultaneously by two independent mass transitions. Experimental raw data were evaluated with the Agilent Mass-Hunter software package, version B10.0. For all metabolites analyzed in this study individual calibration curves were set up and the lower detection limits were determined (see Table S1 + S2, additional file 1). A chromatographic signal was defined as peak when the signal-to-noise ratio exceeded the value of 3.

### Freund’s complete adjuvant induced paw edema model

The experiment was approved by the responsible State animal care committee (Landesamt für Gesundheit und Soziales, Berlin, Germany) and registered under permission number G 0296/18. Mice were kept in groups of three individuals per cage, with free access to food and water, under environmentally controlled conditions (12 h light/dark cycle, light on at 7:00 h; 22–24 °C; humidity 60–65%). Overall, 10 male wildtype and 10 male *Alox15-KI* mice were used in this experiment. Animals were adapted to handling and to the test cages twice a day for 6 consecutive days. On day 7, the basal values for all tests were determined (paw volume, von Frey- and Hargraeves test) and afterwards i. pl. injections were performed. Left hind paw: injection of 20 µl 0.9% NaCl; right hind paw: injection of 20 µl M*. butyricum* in Freund’s complete adjuvant containing 50 µg M*. butyricum*. Animals were monitored on day 8 according to the score sheet established in agreement with the animal welfare administration. On day 9 (2 days after CFA injection), animals were tested in the von Frey and Hargreaves test and the paw volumes were determined. Von Frey and Hargreaves tests were carried out as described in [27].

### Statistics and data presentation

Unless stated otherwise, the experimental raw data were statistically evaluated using the two-way ANOVA function of the GraphPad Prism software package, version 8.4.3 for windows (GraphPad Software, San Diego, USA). For evaluation of the time course (Fig. 2A) the two-way ANOVA repeated measurement function was used. This software package was also employed for generating the images.

## Results

### Alox15 knock-in mice express the arachidonic acid 15-lipoxygenating Leu353Phe Alox15 mutant instead of the arachidonic acid 12-lipoxygenating wildtype enzyme

**Fig. 1:**
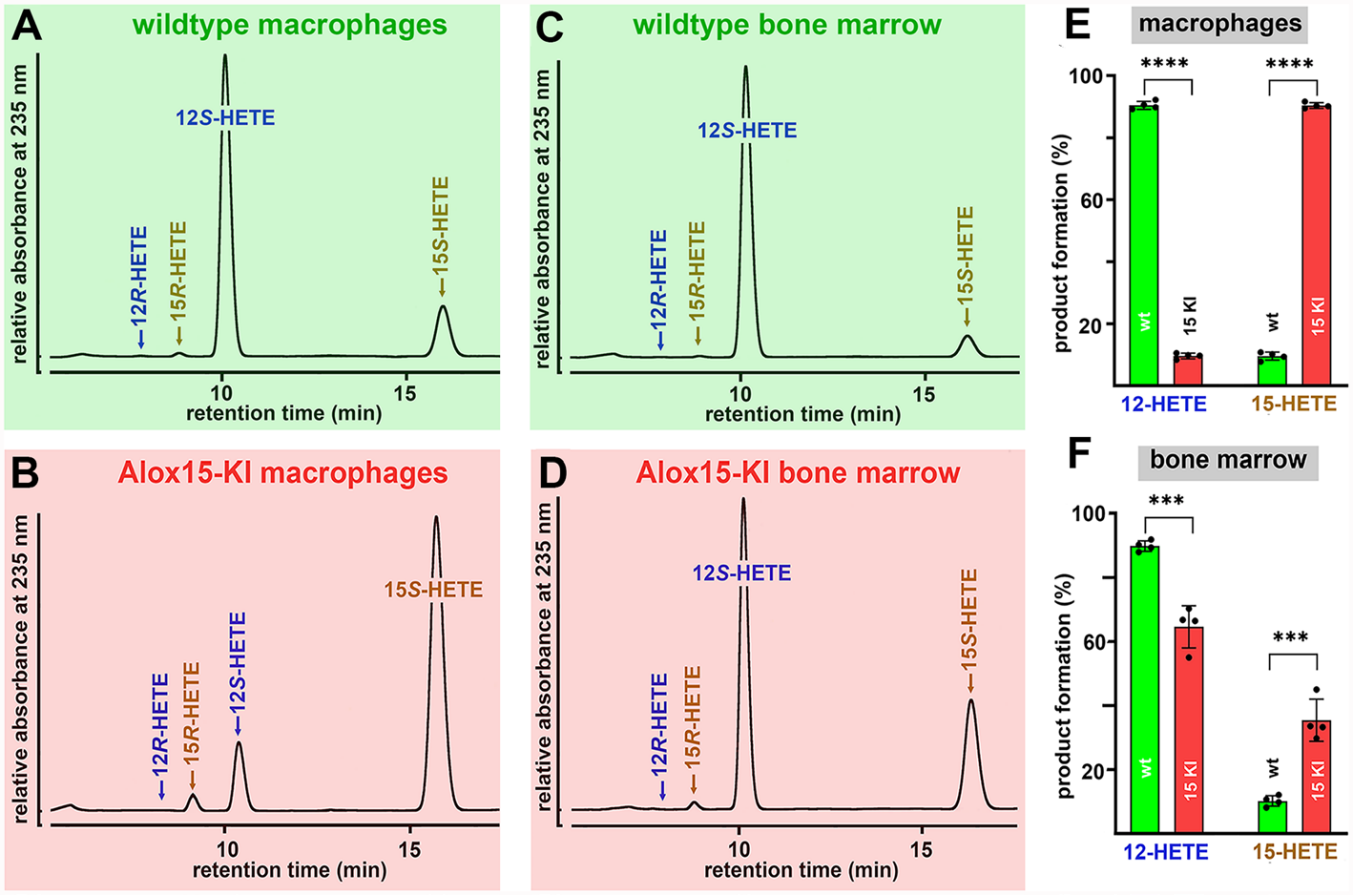
Ex vivo mouse Alox15 activity assays. Peritoneal lavage cells and bone marrow cells were prepared from four different animals of either genotype, ex vivo activity assays were carried out. The AA oxygenation products were prepared by RP-HPLC and further analyzed by combined normal-phase/chiral-phase HPLC. **A** Major AA oxygenation products formed by wildtype peritoneal lavage cells. **B** Major AA oxygenation products formed by *Alox15-KI* peritoneal lavage cells. **C** Major oxygenation products formed by wildtype bone marrow cells. **D** Major oxygenation products formed by *Alox15-KI* bone marrow cells. **E** Quantification of the product patterns formed by peritoneal lavage cells of wildtype and *Alox15-KI* mice and statistical comparison (Mann–Whitney U-test) of the two genotypes (*n* = 4, ****p < 0.0001). **F** Quantification of the product patterns formed by bone marrow cells of wildtype *and Alox15-KI* mice and statistical comparison (Mann–Whitney U-test) of the two genotypes (*n* = 4, ***p < 0.001)

As most mammalian ALOX15 orthologs [28] mouse Alox15 is an AA 12-lipoxygenating enzyme [2, 22] and in vitro mutagenesis studies (Leu353Phe exchange) humanized the reaction specificity of the enzyme [7, 29, 30]. This mutagenesis strategy did also work in vivo since *Alox15-KI* mice which express the Leu353Phe mutant of mouse Alox15 instead of the wildtype enzyme express an AA 15-lipoxygenating enzyme [9]. To confirm these findings for the present study, we prepared peritoneal lavage cells and bone marrow cells from *Alox15-KI* mice and wildtype control animals and performed ex vivo activity assays. From Fig. 1A it can be seen that 12S-HETE was the major AA oxygenation product formed by peritoneal lavage cells of wildtype mice and that the corresponding R-enantiomer was almost absent. 15-HETE was also formed and here again we observed a strong preponderance of the S-isomer. When *Alox15-KI* peritoneal lavage cells were used as enzyme source (Fig. 1B) 15S-HETE was the major AA oxygenation product and 12S-HETE was identified as minor side product. Similar experiments were carried out with the cells of four different individuals of each genotype and statistic evaluation of the product patterns is given in Fig. 1E. According to these data wildtype mouse Alox15 converts AA to a product mixture consisting of 12S-HETE and 15S-HETE in a ratio of 1: 10. In contrast, peritoneal lavage cells of *Alox15-KI* mice form an inverse product mixture. Here the 15S-HETE/12S-HETE ratio was 10: 1. When normalized to equal cell numbers the amounts of AA oxygenation products were similar for both genotypes.

Next, we performed similar ex vivo activity assays with bone marrow cells. In these cells Alox15 is also expressed at high levels but in addition the *Alox12* gene is also active. As expected 12*S*-HETE was identified as dominant AA oxygenation product of wildtype bone marrow cells and small amounts of 15S-HETE were also present (Fig. 1C). When *Alox15-KI* bone marrow cells were used as enzyme source (Fig. 1D) the relative share of 15*S*-HETE was strongly increased (threefold) but 12*S*-HETE remained the major AA oxygenation product. Similar experiments were carried out with the cells of four different individuals of each genotype and statistic evaluation of the product patterns is given in Fig. 1F. The relatively high share of 12*S*-HETE may be related to the high expression of Alox12 in these cells. Since the *Alox12* gene was not affected by our in vivo mutagenesis strategy it contributed to AA oxygenation by these cells. This is not the case for peritoneal lavage cells since Alox12 is hardly expressed in these cells. Taken together the data presented in Fig. 1 indicate that in vivo Leu353Phe exchange functionally humanized mouse Alox15.

### In the DSS colitis model Alox15-KI mice develop less intense inflammatory symptoms

**Fig. 2:**
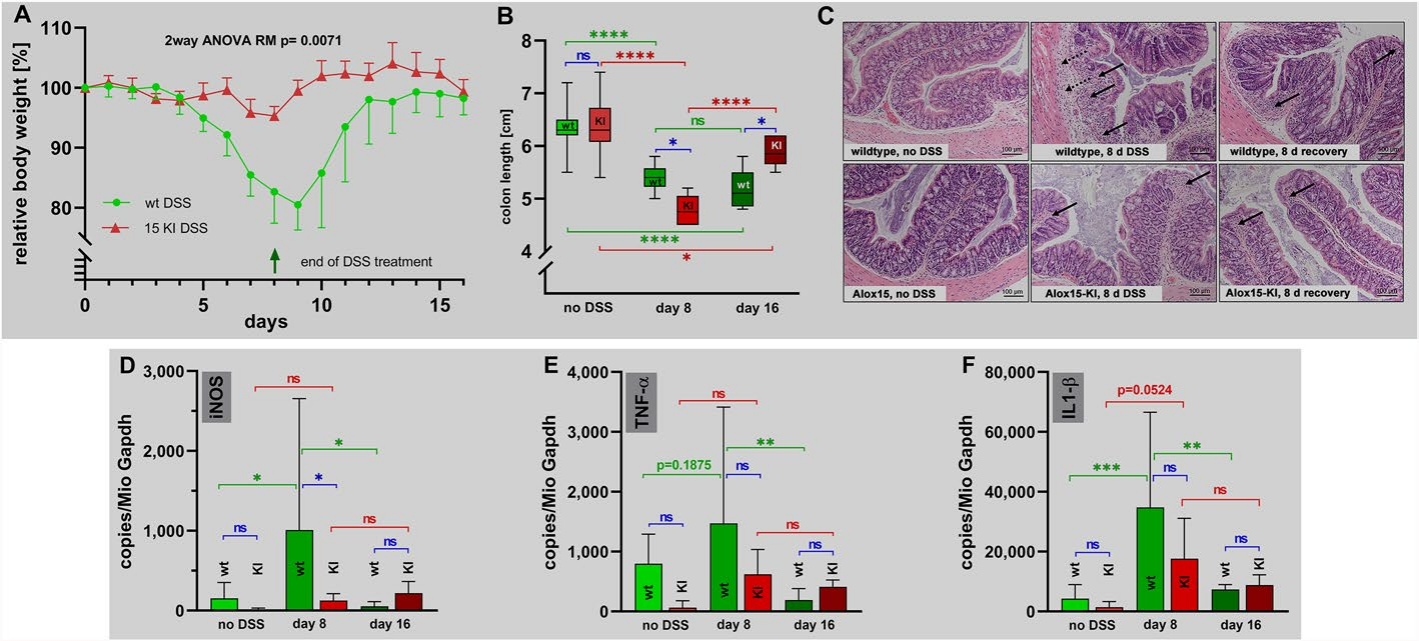
DSS-induced colitis in *Alox15-KI* mice and wildtype control animals. Colonies of *Alox15-KI* mice, which express an Alox15 mutant (Leu353Phe) with humanized reaction specificity, and of wildtype control animals were established (see Materials and Methods) and tested in the DSS-induced experimental colitis model. The experimental approach, animal grouping and quantification of the readout parameters are explained in detail in Materials and Methods. **A** Bodyweight kinetics of *Alox15-KI* mice and of wildtype controls. **B** Colon length determined at different time points of the experimental protocol. **C** Representative histological cross section of the colon at different time points of the experimental protocol. Upper panels, wildtype mice, lower panels, *Alox15-KI* mice. Solid arrows indicate infiltrations of inflammatory cells. Dotted arrows indicate mucosal ulcerations. **D**–**F** Expression profiles of classical pro-inflammatory gene products. At different time points of DSS treatment animals were sacrificed, the colon was prepared and total RNA was extracted. Aliquots of the RNA preparations were reversely transcribed and qRT-PCR was carried out to quantify the colonic steady state mRNA concentrations. These concentrations are given by the copy numbers of target mRNA per 10^6^ copies of Gapdh mRNA. Experimental raw data were evaluated statistically with the two-way ANOVA function of the GrahPad Prism program and the following n-numbers were included: wildtype (wt) animals without DSS, *n* = 5; *Alox15-KI* (15-KI) animals without DSS, *n* = 5; wildtype (wt) animals 8 days DSS, *n* = 5; *Alox15-KI* (15-KI) animals 8 days DSS, *n* = 5; wildtype (wt) animals 8 days after DSS removal, n = 5; *Alox15-KI* (KI) animals 8 days after DSS removal, *n* = 5. *ns* statistically not significant. *p < 0.05, **p < 0.01, ***p < 0.001, ****p < 0.0001

The DSS-colitis model is a frequently employed model of intestinal inflammation [23, 24]. Since humanization of Alox15 reaction specificity impacts the in vitro biosynthetic capacity of the enzyme for pro-resolving lipoxins [31, 32] we tested the susceptibility of *Alox15-KI* mice in the DSS colitis model. Female mice were treated with 1.5% DSS in the drinking water for 8 consecutive days. Then the DSS solution was replaced with normal drinking water and the animals were allowed to recover for 8 days. When wildtype animals were challenged this way (green symbols in Fig. 2A) we observed that at day 5 the animals started losing body weight. At day 9 the lowest body weights were reached and the animals started to recover. At day 16 the original body weights were reached again. For *Alox15-KI* mice different body weight kinetics were recorded (red symbols in Fig. 2A). For these mice the body weights remained unchanged between day 0–6. At day 7 and 8 the body weights were slightly reduced but already at day 9 the animals returned to their original body weights. These body weight kinetics suggested that *Alox15-KI* mice were strongly protected from DSS-induced enteral inflammation.

As second clinical readout parameter we determined the degree of colon shrinkage [23, 24]. Here we found that DSS treatment of wildtype mice induced significant colon shortening (green symbols in Fig. 2B) but the colon lengths did not normalize during the recovery period. For the *Alox15-KI* mice we also observed significant colon shrinkage but after the recovery period the original colon lengths were almost reached again. Interestingly, the degree of colon shrinkage was more pronounced in *Alox15-KI* mice and this data suggests that humanization of Alox15 promoted colon shrinkage. However, after the recovery period the colon lengths of the *Alox15-KI* mice did almost reach the original values (day 0) and this data suggests that *Alox15-KI* mice apparently recovered more rapidly after removal of the inflammatory stimulus.

Next, we evaluated histological preparations of colon tissue for inflammatory symptoms and quantified three morphological inflammation parameters according to a semi-quantitative scoring system [33]: (1) extent of neutrophil infiltration, (2) extent of epithelial defects, (3) extent of mucosal ulcerations. For both, *Alox15-KI* mice and wildtype control animals we observed normally structured colon walls before DSS application (Fig. 2C, left panels). Thus, humanization of the Alox15 did not impact the structure of the colon wall. After 8 days of DSS treatment we observed significant neutrophil infiltrations in the colon wall of both *Alox15-KI* mice and wildtype controls but according to our scoring system the intensity of the inflammatory alterations was higher in the wildtype mice. During the resolution period most of the inflammatory alterations disappeared but we still observed neutrophils in the colon wall of both genotypes. The quantitative results of the scoring procedure (see Table S3, additional file 1) indicate that our experimental protocol induced a rather mild experimental colitis.

Intestinal inflammation alters the gene expression patterns in the colon wall and we profiled by qRT-PCR (Fig. 2D–F) the expression of three independent pro-inflammatory gene products [inducible nitric oxide synthase (iNOS), tumor necrosis factor alpha (TNFalpha) and interleukin-1beta (IL1beta)]. We found that these genes exhibited similar expression kinetics during enteral inflammation. Their expression levels were low before DSS administration but increased during the acute phase of inflammation. After the recovery period the expression levels were down to starting values. In principle, similar kinetics were observed for wildtype control animals and *Alox15-KI* mice but there were interesting differences between the two genotypes. At day 8 of DSS treatment iNOS expression was (Fig. 2D) significantly higher in wildtype mice than in *Alox15-KI* animals but similar differences were not observed in normal colon tissue (no DSS) and after the recovery period (day16). Considering the fact that iNOS is a classical pro-inflammatory enzyme, the lower expression levels of this enzyme in *Alox15-KI* mice at day 8 of DSS treatment were consistent with the protective effect of Alox15 humanization shown in Fig. 2A. Similar results were obtained for TNFalpha (Fig. 2E) and IL1beta (Fig. 2F) but for these proteins the differences did not reach the level of statistical significance. The most interesting finding of our expression studies was that during the acute phase of inflammation the expression of iNOS, TNFalpha and IL1beta were upregulated in wildtype mice. In contrast, in *Alox15-KI* mice the degree of upregulation was less pronounced.

### Profiles of specific oxylipins in colon tissues of Alox15-KI mice and wildtype controls during the time-course of DSS colitis

**Fig. 3:**
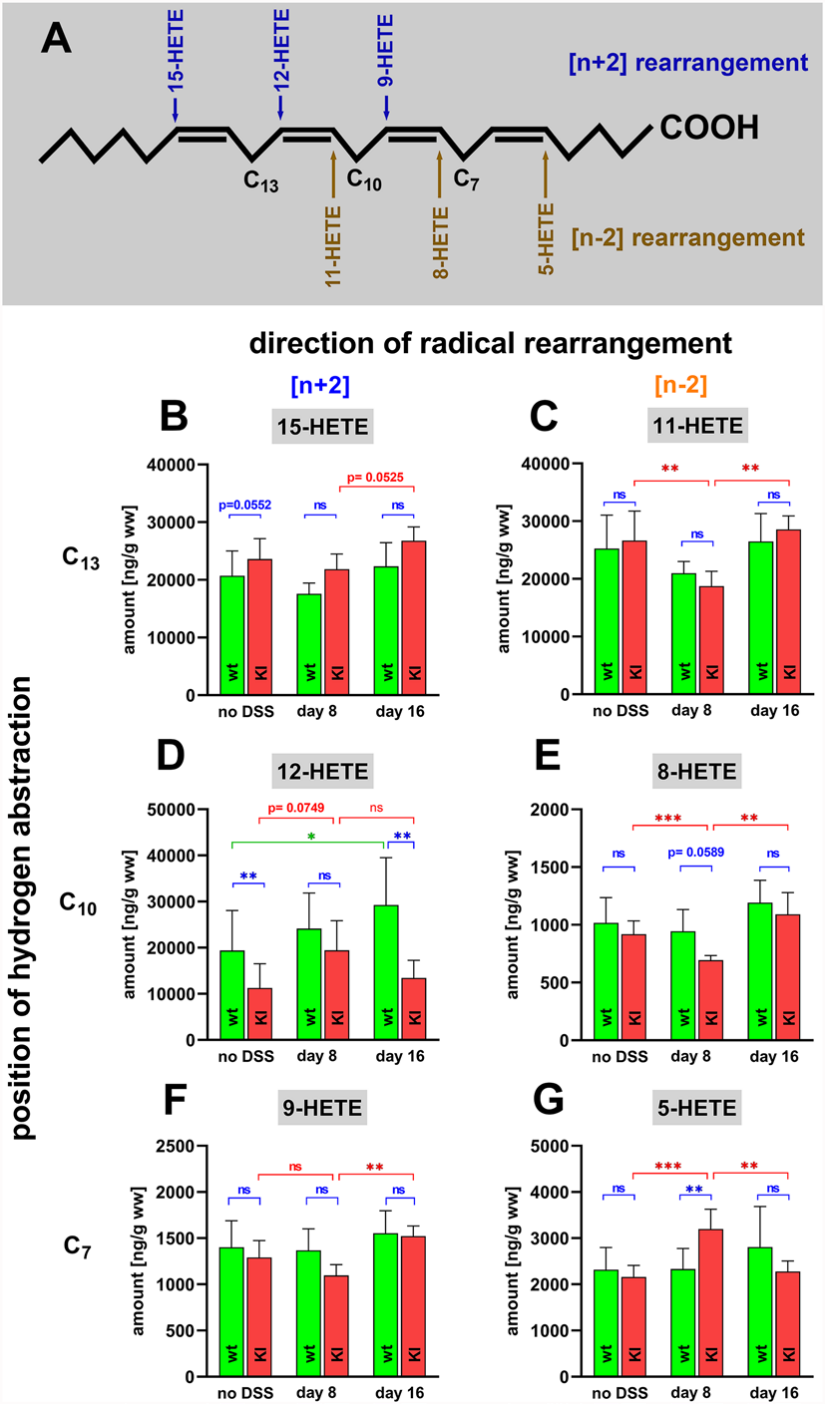
Quantification of arachidonic acid oxygenation products in colon tissue at different time points of DSS-induced colitis. Colitis induction, sample workup and LC–MS/MS analysis as described in Materials and Methods. **A** AA is oxidized to six major oxygenation products (HETE-isomers). Biosynthesis of 15-HETE, 12-HETE and 9-HETE involve hydrogen abstraction from C_13_, C_10_ and C_7_, respectively, as well as [+ 2] radical rearrangement (blue). Formation of 11-HETE, 8-HETE and 5-HETE proceeds via C_13_, C_10_ and C_7_, respectively, hydrogen abstraction and [-2] radical rearrangement (brown). **B** Colonic 15-HETE concentrations. **C** Colonic 11-HETE concentrations, **D** Colonic 12-HETE concentrations, **E** Colonic 8-HETE concentrations, **F** colonic 9-HETE concentrations. **G** Colonic 5-HETE concentrations. The experimental raw data were evaluated statistically with the two-way ANOVA function of the GraphPad Prism program and the following n-numbers were included: wildtype (wt) animals without DSS, *n* = 5; *Alox15-KI* (KI) animals without DSS, *n* = 5; wildtype (wt) animals 8 days DSS, *n* = 5; *Alox15-KI* (KI) animals 8 days DSS, *n* = 5; wildtype (wt) animals 8 days after DSS removal, *n* = 5; *Alox15-KI* (KI) animals 8 days after DSS removal, *n* = 5. *ns* not significant. *—*p* < 0.05, **—*p* < 0.01, ***—*p* < 0.001

AA is one of the major polyenoic fatty acids in mammalian cells and there are six major AA oxygenation products (15-HETE, 12-HETE, 11-HETE, 9-HETE, 8-HETE, 5-HETE). These products are formed via nonenzymatic oxygenation reactions but also via the ALOX pathway of the AA cascade. Their biosynthetic mechanisms (Fig. 3A) involve hydrogen abstraction from one of the three bisallylic methylenes (C_7_, C_10_, C_13_) and either [+ 2] (blue) or [-2] (brown) radical rearrangement.

For 11-HETE (Fig. 3C), 9-HETE (Fig. 3F) and 8-HETE (Fig. 3E) we did not observe significant differences between the two genotypes at either time point of the enteral inflammation. If Alox15 significantly contributes to the formation of HETE isomers in the noninflamed colon wall we expected reduced 12-HETE levels in the colon tissue of *Alox15-KI* mice when compared with wildtype controls. In contrast, the colonic 15-HETE levels should be elevated. Our oxylipidomic data confirmed that the 12-HETE concentrations were higher in wildtype mice (Fig. 3D) although the difference between the two genotypes at day 8 of DSS treatment did not reach the level of statistical significance. Unfortunately, we did not observe an anti-parallel decrease in the 15-HETE levels (Fig. 3B). As leukotriene B4 (LTB4) 5-HETE is an AA oxygenation product formed via the Alox5 pathway. Since Alox5 is a canonic pro-inflammatory enzyme colonic 5-HETE concentrations should be elevated at day 8 of the experimental protocol. Such kinetics were indeed observed for *Alox15-KI* mice (red bars in Fig. 3G). However, for wildtype mice we did not observe similar kinetics. Here no significant differences were observed between the different time points of the inflammatory process.

**Fig. 4:**
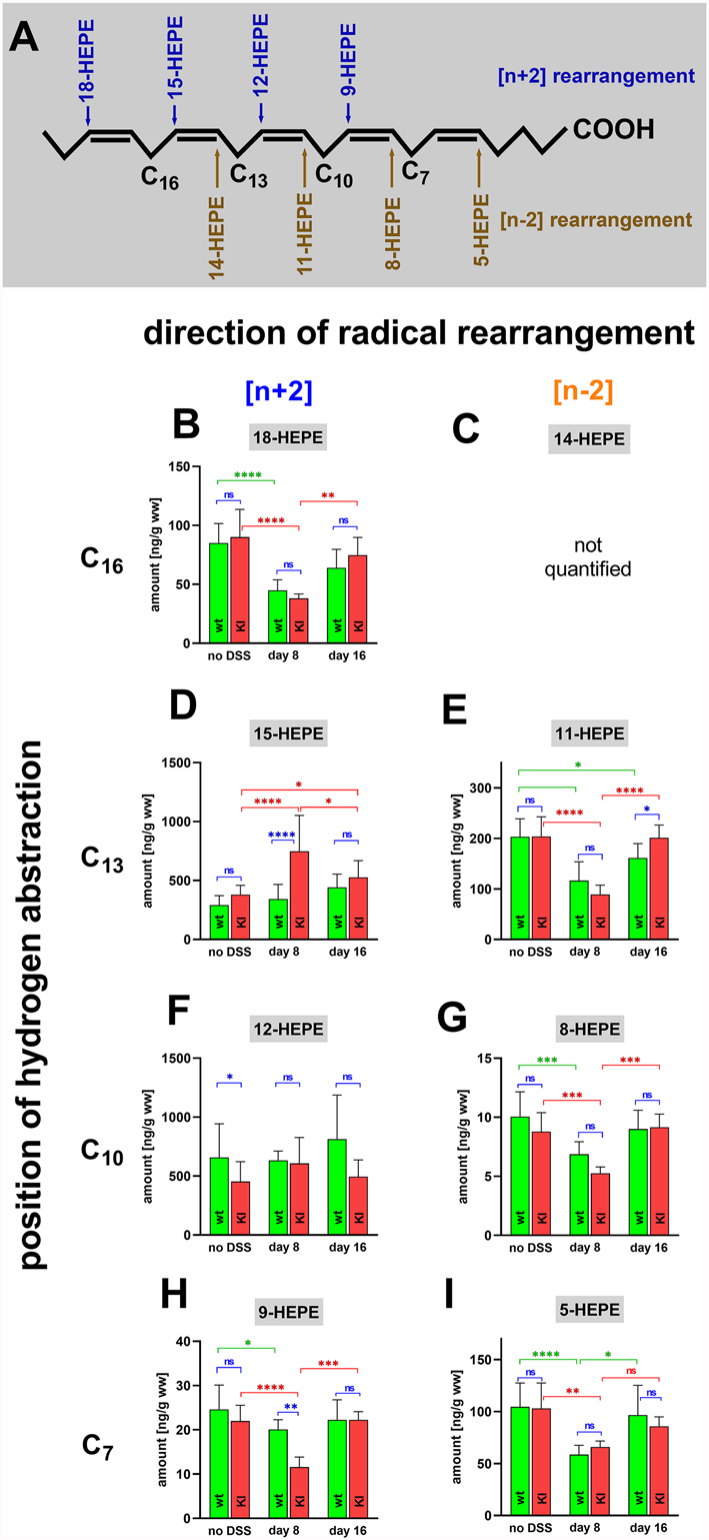
Quantification of eicosapentaenoic acid oxygenation products in colon tissue at different time points of DSS-induced colitis. Colitis induction, sample workup and LC–MS/MS analysis as described in Materials and Methods. **A** EPA is oxidized to eight major oxygenation products (HEPE-isomers). Biosynthesis of 18-HEPE, 15-HEPE, 12-HEPE and 9-HEPE involve hydrogen abstraction from C_16_, C_13_, C_10_ and C_7_, respectively, as well as [+ 2] radical rearrangement (blue). Formation of 14-HEPE, 11-HEPE, 8-HEPE and 5-HEPE proceeds via C_16_, C_13_, C_10_ and C_7_, respectively, hydrogen abstraction and [-2] radical rearrangement (brown). **B** Colonic 18-HEPE concentrations. **C** Colonic 14-HEPE concentrations was not measured. **D** Colonic 15-HEPE concentrations. **E** Colonic11-HEPE concentrations. **F** colonic 12-HEPE concentrations. **G** Colonic 8-HEPE concentrations. **H** Colonic 9-HEPE concentrations. **I** Colonic 5-HEPE concentrations. The experimental raw data were evaluated statistically with the two-way ANOVA function of the GraphPad Prism program and the following n-numbers were included: wildtype (wt) animals without DSS, *n* = 5; *Alox15-KI* (KI) animals without DSS, *n* = 5; wildtype (wt) animals 8 days DSS, *n* = 5; *Alox15-KI* (KI) animals 8 days DSS, n = 5; wildtype (wt) animals 8 days after DSS removal, *n* = 5; *Alox15-KI* (KI) animals 8 days after DSS removal, *n* = 5. *ns* not significant, *—*p* < 0.05, **—*p* < 0.01, ***—*p* < 0.001, ****—*p* < 0.0001

**Fig. 5:**
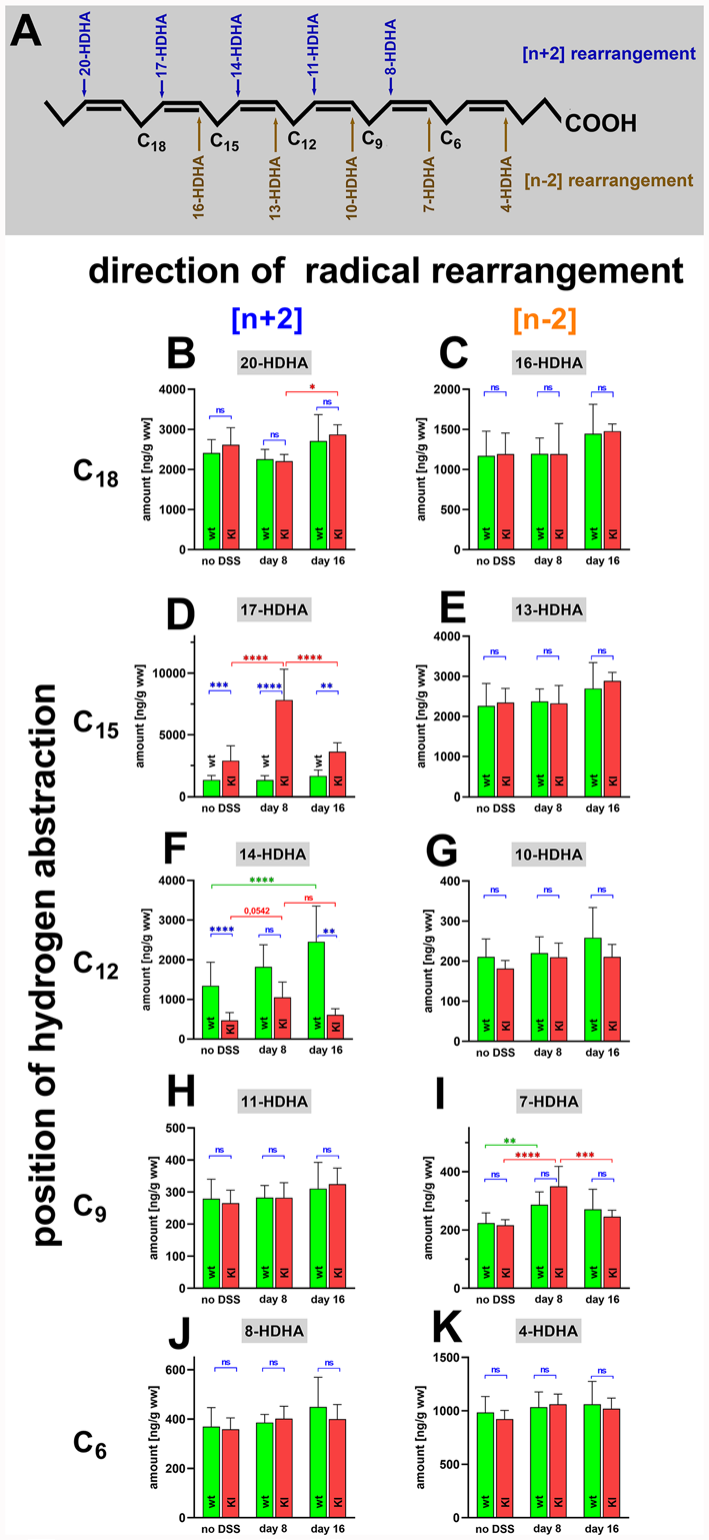
Quantification of docosahexaenoic acid oxygenation products in colon tissue at different time points of DSS-induced colitis. Colitis induction, sample workup and LC–MS/MS analysis as described in Materials and Methods. **A** DHA is oxidized to ten major oxygenation products (HDHA-isomers). Biosynthesis of 20-HDHA, 17-HDHA, 14-HDHA, 11-HDHA, and 8-HDHA involves hydrogen abstraction from C_18_, C_15_, C_12,_ C_9_ and C_6_, respectively, as well as [+ 2] radical rearrangement (blue). Formation of 16-HDHA, 13-HDHA, 10-HDHA, 7-HDHA, and 4-HDHA proceeds via C_18_, C_15_, C_12_ and C_9_ and C_6_, respectively, hydrogen abstraction and [-2] radical rearrangement (brown). **B** Colonic 20-HDHA concentrations. **C** Colonic 16-HDHA. D) Colonic 17-HDHA concentrations. **E** Colonic 13-HDHA concentrations. **F** colonic 14-HDHA concentrations. **G** Colonic 10-HDHA concentrations. **H** Colonic 11-HDHA concentrations. **I** Colonic 7-HDHA concentrations. **J** Colonic 8-HDHA concentrations, **K** 4-HDHA concentrations. The experimental raw data were evaluated statistically with the two-way ANOVA function of the GraphPad Prism program and the following n-numbers were included: wildtype (wt) animals without DSS, *n* = 5; *Alox15-KI* (KI) animals without DSS, *n* = 5; wildtype (wt) animals 8 days DSS, *n* = 5; *Alox15-KI* (KI) animals 8 days DSS, *n* = 5; wildtype (wt) animals 8 days after DSS removal, *n* = 5; *Alox15-KI* (KI) animals 8 days after DSS removal, *n* = 5. *ns* not significant, *—*p* < 0.05, **—*p* < 0.01, ***—p < 0.001, ****—*p* < 0.0001

Next, we quantified the oxygenation products of 5,8,11,14,17-eicosapentaenoic acid (EPA). This PUFA carries 4 bisallylic methylenes and thus (Fig. 4A), 8 different major EPA oxygenation products can be formed. Except for 14-HEPE (Fig. 4C), which was not part of our analytical protocol, we quantified the colon concentrations of these metabolites but did not detect significant differences between the two genotypes for 18-HEPE (Fig. 4B), 8-HEPE (Fig. 4G) and 5-HEPE (Fig. 4I) at any time point of the inflammatory process. For 11-HEPE (Fig. 4E) we observed a small but significant difference between the two genotypes at the end of the recovery period. 15-HEPE (Fig. 4D) and 12-HEPE (Fig. 4F), which are the major EPA oxygenation products formed by mouse Alox15 and its Leu353Phe mutant, were detected as dominant EPA oxygenation products in the colon wall. In contrast, 8-HEPE (Fig. 4G) and 9-HEPE (Fig. 4H) were only present at minor concentrations.

Recombinant mouse Alox15 oxygenates EPA in vitro predominantly to 12-HEPE but its functionally humanized Leu353Phe mutant forms a 2: 1 mixture of 15-HEPE and 12-HEPE [4]. If our genetic manipulation of the *Alox15* gene is mirrored on the level of colon lipids, *Alox15-KI* mice were expected to show elevated 15-HEPE levels but reduced 12-HEPE levels when compared with wildtype control animals. When we quantified the 15-HEPE tissue concentrations of *Alox15-KI* mice and corresponding wildtype controls at day 8 of DSS treatment we indeed observed significantly elevated levels in *Alox15-KI* mice (Fig. 4D). A similar trend was also observed at the other time points of enteral inflammation (no DSS, day 16) but here the differences did not reach the level of statistical significance. Unfortunately, we did not see an anti-parallel decrease in the 12-HEPE levels in *Alox15-KI* mice. In fact, no significant differences in the colonic 12-HEPE levels were detected when the two genotypes were compared (Fig. 4F).

Because of its high degree of unsaturation 4,7,11,13,16,19-docosahexaenoic acid (DHA) is very sensitive to auto-oxidation and 10 different cis–trans conjugated diene products (HDHA-isomers) can be formed (Fig. 5A). Moreover, this n-3 polyenoic fatty acid is a suitable substrate for different ALOX-isoforms which convert this substrate to highly specific oxygenation products [4].

When we compared the colonic HDHA concentrations at the different time points of the experimental protocol we did not observe significant differences between the two genotypes for 20-HDHA (Fig. 5B), 16-HDHA (Fig. 5C), 13-HDHA (Fig. 5E), 10-HDHA (Fig. 5G), 11-HDHA (Fig. 5H), 7-HDHA (Fig. 5I), 8-HDHA (Fig. 5J) and 4-HDHA (Fig. 5K). In contrast, for the Alox15 products 17-HDHA (Fig. 5D) and 14-HDHA (Fig. 5F) we detected significant differences between humanized *Alox15-KI* mice and wildtype control animals. Recombinant mouse Alox15 oxygenates DHA to a 1:2 mixture of 17- and 14-HDHA [4]. If our genetic manipulation of the *Alox15* gene is mirrored on the level of the colon lipids, *Alox15-KI* mice should have lower tissue concentrations of 14-HDHA but elevated levels of 17-HDHA when compared with wildtype controls. Our colon oxylipidome analyses revealed that *Alox15-KI* mice (Fig. 5D) show elevated 17-HDHA tissue concentrations when compared with wildtype controls independent of the time point of the disease. In contrast, the 14-HDHA concentrations in the colon of wildtype animals were higher than those in the *Alox15-KI* mice (Fig. 5F) although at day 8 the difference did not reach the level of statistical significance. When we explored the kinetic of 17-HDHA in *Alox15-KI* mice during the time course of experimental inflammation (Fig. 5D) we found significantly elevated tissue levels after 8 days of DSS treatment. After the resolution period the 17-HDHA levels were back to the initial levels. However, when we followed the 17-HDHA kinetics in wildtype mice we did not see such kinetic differences (Fig. 5D). These data suggest that the catalytic activity of the mutant Alox15 may play a role in the pathogenesis of DSS colitis. Similar kinetics were also observed for the colon concentrations of 7-HDHA, the major DHA oxygenation product of mouse Alox5 (Fig. 5I) but for this metabolite we did not observe significant differences between the two genotypes.

**Fig. 6:**
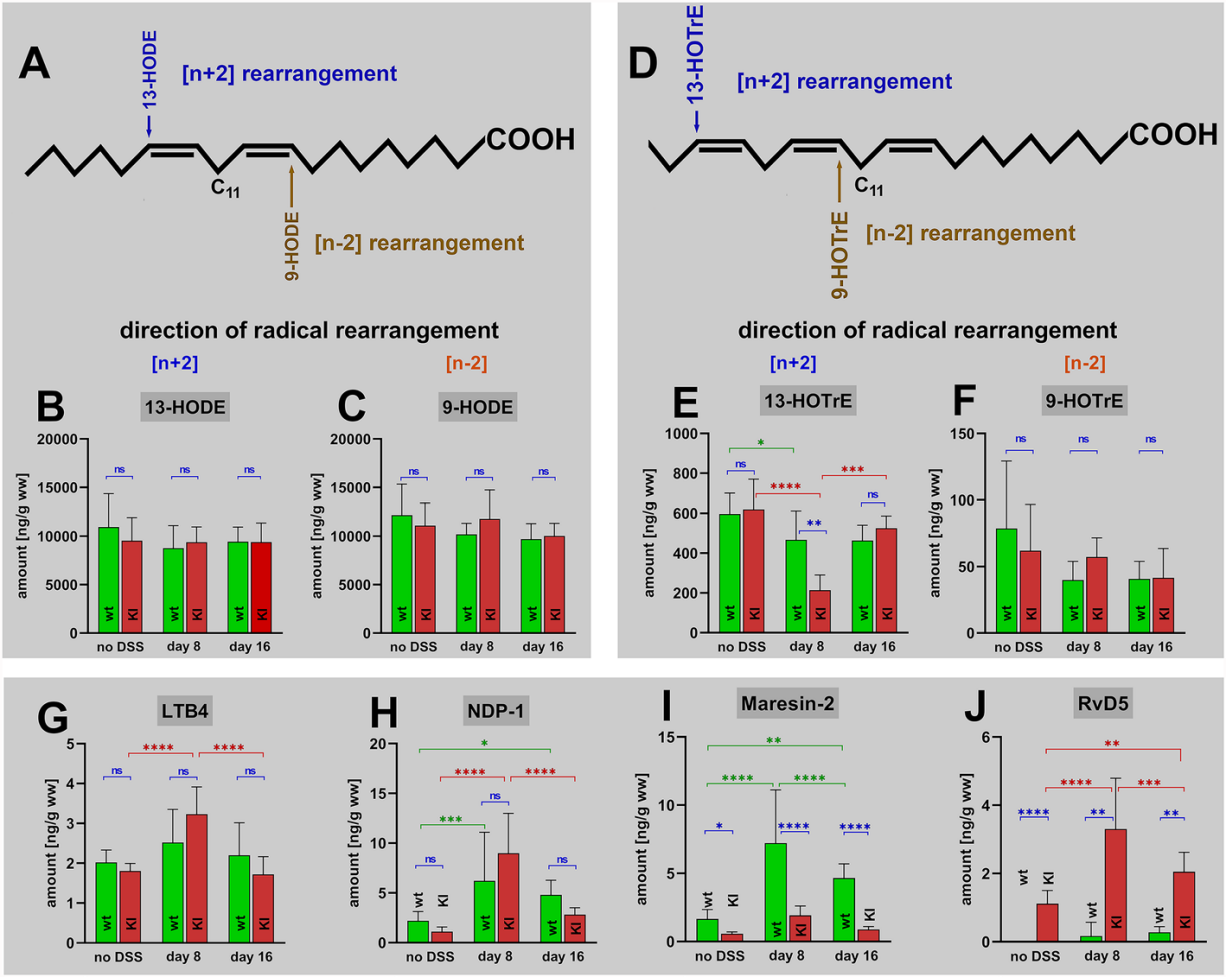
Quantification of the oxygenation products of different polyenoic fatty acids in colon tissue at different time points of DSS-induced colitis. Colitis induction, sample workup and LC–MS/MS analysis as described in Materials and Methods. **A** LA is oxidized to two major oxygenation products (HODE-isomers). Biosynthesis of both 9-HODE and 13-HODE involves hydrogen abstraction from C_11_ but either [+ 2] (13-HODE, blue) or [-2] (9-HODE, brown) radical rearrangement. **B** Colonic concentrations of 13-HODE. **C** Colonic concentrations of 9-HODE. **D** ALA carries two bisallylic methylenes (C_11_, C_14_) and thus, four major oxygenation products (13-HOTrE, 9-HOTrE, 16-HOTrE, 12-HOTrE) can be formed but we only profiled the colon concentrations of 13- and 9-HOTrE. Biosynthesis of these two metabolites involves hydrogen abstraction from C_11_ but either [+ 2] (13-HODE, blue) or [-2] (9-HODE, brown) radical rearrangement. **E** Colonic concentrations of 13-HOTrE. **F** Colonic concentrations of 9-HOTrE. **G** Colonic concentrations of leukotriene B_4_. **H** Colonic concentrations of neuroprotection-1. **I** Colonic concentrations of maresin-1. **J** Colonic concentrations of resolvin D_5_. The experimental raw data were evaluated statistically with the two-way ANOVA function of the GraphPad Prism program and the following n-numbers were included: wildtype (wt) animals without DSS, *n* = 5; *Alox15-KI* (15-KI) animals without DSS, *n* = 5; wildtype (wt) animals 8 days DSS, *n* = 5; *Alox15-KI* (15-KI) animals 8 days DSS, *n* = 5; wildtype (wt) animals 8 days after DSS removal, *n* = 5; *Alox15-KI* (15-KI) animals 8 days after DSS removal, *n* = 5. *ns* statistically not significant. *—*p* < 0.05, **—*p* < 0.01, ***—*p* < 0.001, ****—*p* < 0.0001

Linoleic acid (LA) and alpha-linolenic acid (ALA) are among the most abundant polyenoic fatty acids in mammalian cells. LA involves only one (Fig. 6A) bisallylic methylene and thus, only two major cis–trans conjugated dienes (13-HODE, 9-HODE) can be formed. Recombinant mouse Alox15 converted LA to 13-HODE but this is also the case for its functionally humanized Leu353Phe mutant. When we analyzed the colon concentrations of 13-HODE(Z,E) (Fig. 6B) and 9-HODE(E,Z) (Fig. 6C), we did not detect significant differences between *Alox15-KI* mice and wildtype controls. Moreover, 13-HODE and 9-HODE were present at similar concentrations (Fig. 6B + C) and these data suggest that LA auto-oxidation is the dominant biosynthetic pathway. Moreover, the colon concentrations of the 13-HODE and 9-HODE did not change during the time course of inflammation (Fig. 6B + C) suggesting that these metabolites may not be of major patho-physiological relevance for colon inflammation.

For the two ALA (Fig. 6D) metabolites 13-HOTrE(Z,E,Z) and 9-HOTrE(E,Z,Z) (Fig. 6E + F) similar conclusions can be drawn but there were two interesting peculiarities: (i) 13-HOTrE(Z,E,Z) was detected in much higher quantities than 9-HOTrE(E,Z,Z) (Fig. 6E + F), which excludes auto-oxidation as major biosynthetic pathway. (ii) At day 8 of the DSS treatment we analyzed significantly lower 13-HOTrE(Z,E,Z) concentrations in the colons of *Alox15-KI* mice when compared with wildtype controls. Since the biosynthetic route of 13-HOTrE(Z,E,Z) formation during colitis remains unclear the reduction of 13-HOTrE(Z,E,Z) at the peak of inflammation is difficult to interpret.

Finally, we quantified the colon concentrations of canonic pro- and anti-inflammatory lipid mediators. LTB4 is a classical pro-inflammatory eicosanoid [34], which should be present in the colon tissue at elevated concentrations at the peak of inflammation. When we profiled the colonic LTB4 levels during the time course of enteral inflammation we observed the expected kinetics but the differences between the three time points in wildtype mice did not reach the level of statistical significance (Fig. 6G). In the *Alox15-KI* mice we observed similar kinetics but here the differences between the three time points were statistically significant (Fig. 6G). When we compared the colonic LTB4 levels between *Alox15*-KI mice and wildtype controls we did not detect significant differences between the two genotypes at either time point. (Fig. 6G). Thus, our genetic manipulation of the *Alox15* gene did obviously not alter LTB4 metabolism.

Neuroprotectin 1 (NPD1, 10*R*,17*S*-dihydroxy-4*Z*,7*Z*,11*E*,13*E*,15*Z*,19*Z*-docosahexaenoic acid) is a dihydroxy derivative of DHA, exhibits neuroprotective properties [35] and has been classified as special pro-resolving mediator (SPM) [36]. We detected small amounts of NPD-1 in the colon of untreated wildtype mice (Fig. 6H) but the tissue concentrations were strongly increased at day 8 of DSS-treatment. After the recovery period the NPD-1 levels went back to normal. For *Alox15-KI* mice similar kinetics were observed but we did not detect significant differences between the two genotypes at either time point of the experimental colitis. Almost identical kinetics were measured for the dihydroxy SPMs maresin-2 (Mar-2, 13R,14S-dihydroxy-4Z,7Z,9E,11E,16Z,19Z-docosahexaenoic acid, Fig. 6I) and resolvin D5 (RvD5, 7S,17S-dihydroxy-4Z,8E,10Z,13Z,15E,19Z-docosahexaenoic acid, Fig. 6J). There were, however, interesting differences in the colon concentrations of these two SPMs. For Mar-2 we measured consistently lower concentrations in the colon tissue of *Alox15-KI* mice (Fig. 6I). These differences, which were statistically significant, suggest that humanization of the reaction specificity of mouse Alox15 may have impaired the biosynthetic capacity of the enzyme for maresin-2. The possible mechanistic basis for this finding will be explained later on in this paper (Discussion). In contrast, the biosynthetic capacity for RvD5 was apparently augmented by humanization of the reaction specificity of mouse Alox15. For this metabolite we consistently measured significantly higher colon concentrations in the colon tissue of *Alox15-KI* mice. These results can easily be explained by the modified catalytic properties of the humanized Alox15 (see Discussion).

In addition to these dihydroxy SPMs we also attempted to quantify the following representatives of the SPM family: RvD1, RvD2, RvD3, RvD4, LxA4, and LxB4. Unfortunately, neither of these metabolites could be quantified in normal or inflamed colon tissue of either genotype (see Figure S1, additional file 1). If present, their tissue concentrations were below the detection limits of our analytical procedure (see Table S1 + S2, additional file 1).

### There was no difference between Alox15-knock-in mice and wildtype controls in the paw edema inflammation model

**Fig. 7:**
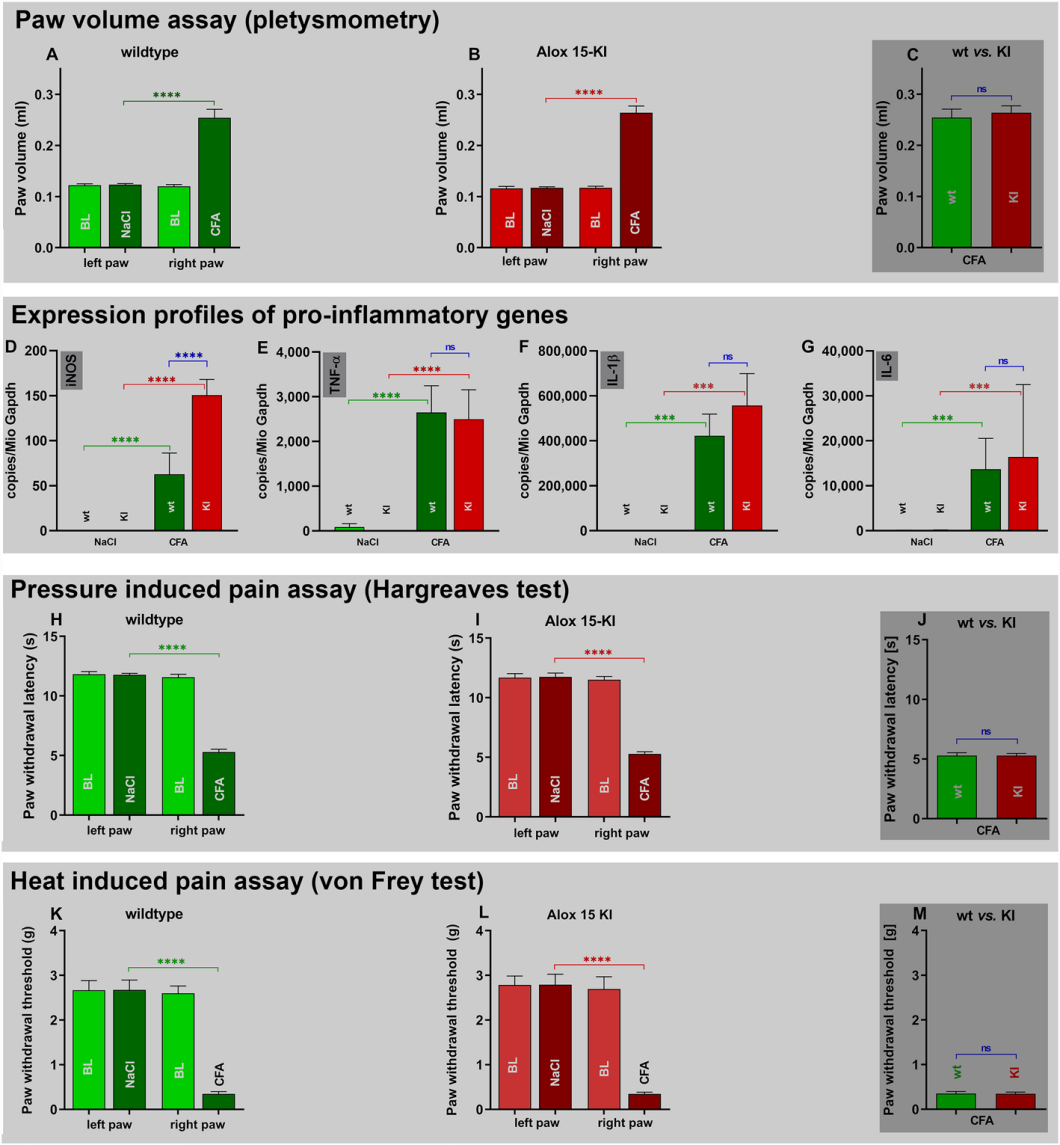
Freund’s complete adjuvant induced paw edema inflammation model. Paw edema was induced by subcutaneous injection of Freund’s complete adjuvant (see Material and Methods. **A**–**C** After 2 days the paw volume was measured as clinical readout parameter for the intensity of the inflammatory reaction. **A** Comparison of paw volumes in wildtype mice. **B** Comparison of paw volumes in *Alox15-KI* mice, **C** Comparison of paw volumes in wildtype vs. *Alox15-KI* mice 2 days after CFA injection. **D**–**G** Quantification of the expression profiles of pro-inflammatory genes by qRT-PCR. **D** iNOS, **E** TNFalpha. **F** IL1beta, **G** IL6. **H**–**J** Two days after CFA injection the paw withdrawal latency (Hargreaves test) was measured as readout parameter for pressure-induced pain perception. **H** Wildtype mice. **I** *Alox15-KI* mice. **J** Comparison of paw withdrawal latency of wildtype *vs. Alox15-KI* mice 2 days after CFA injection. **K**–**M** Two days after CFA injection the paw withdrawal threshold (von Fey test) was measured as readout parameter for heat-induced pain perception. **K** Wildtype mice. **L** *Alox15-KI* mice. **M** Comparison of paw withdrawal threshold of wildtype *vs. Alox15-KI* mice 2 days after CFA injection. The experimental raw data were evaluated statistically with the two-way ANOVA function of the GraphPad Prism program, *n* = 10 in each experimental group. *ns* not significant; ***p < 0.001, ****p < 0.0001

To test the *Alox15-KI* mice in a second type of inflammation we employed the Freund’s complete adjuvant (CFA) induced paw edema model [37]. As clinical readout parameter we determined the degree of paw swelling, the expression of classical pro-inflammatory proteins and the sensitivity of the inflamed paw for thermic (Hargreaves test) and mechanic (von Frey test) stimulation. From Fig. 7A it can be seen that injection of PBS into the hind paw did not alter the paw volume when compared with an untreated paw (blank, BL) of wildtype mice. However, injection of Freund’s complete adjuvant led to a significant increase in the paw volume indicating the formation of an inflammatory edema. Similar alterations were induced in *Alox15-KI* mice (Fig. 7B). When we compared the degree of paw edema of *Alox15-KI* mice and wildtype controls we did not observe significant differences (Fig. 7C). These data suggest that *Alox15-KI* mice are neither protected from nor sensitized to adjuvant induced paw swelling.

Next, we quantified by qRT-PCR the expression of classical pro-inflammatory gene products. As indicated in Fig. 7D iNOS mRNA was absent in normal paw tissue of both *Alox15-KI* mice and wildtype controls. In contrast, 2 days after CFA injection significant levels of iNOS mRNA were detected in the inflamed tissue of both genotypes. We even observed significantly higher iNOS mRNA levels in *Alox15-KI* mice when compared with wildtype control animals suggesting a higher degree of inflammation in this genotype. For TNF alpha, (Fig. 7E), IL1beta (Fig. 7F) and IL6 (Fig. 7G) we obtained similar data but for these pro-inflammatory cytokines we did not observe any differences between *Alox15-KI* mice and wildtype controls. Taken together, these expression profiles are consistent with the previous observation (Fig. 7C) that functional humanization of mouse Alox15 does not protect the animals in this particular inflammation model.

Pain is a frequent symptom of most inflammatory reactions and inflammatory pain can be quantified by a number of complex assay systems. For our study we used the Hargreaves [38] and the von Frey-tests [39] to compare the pain sensitivity of the *Alox15-KI* mice with wildtype control animals. Injection of CFA significantly reduced the paw withdrawal latency (Hargreaves test) of wildtype control mice (Fig. 7H). A similar effect was observed for the *Alox15-KI* mice (Fig. 7I) but we did not detect a significant difference when the two genotypes were compared (Fig. 7J). Thus, functional humanization of mouse Alox15 does not impact the sensitivity of mice for heat induced pain.

When the animals of the two genotypes were tested in the von Frey assay (mechanic pain induction) we found that injection of CFA significantly increased the pain sensitivity of wildtype animals (Fig. 7K). A similar effect was observed in *Alox15-KI* mice (Fig. 7L) but we did not see a significant difference between the two genotypes (Fig. 7M). Here again, humanization of the reaction specificity of mouse Alox15 does not impact the sensitivity of mice against inflammation induced pain.

Taken together the data obtained in the paw edema experiment indicate that humanization of the reaction specificity of mouse Alox15 did hardly impact the inflammatory reaction induced by local administration of Freund’s complete adjuvant.

## Discussion

### Alox15-KI mice are protected in the DSS colitis model but not in the Freund’s complete adjuvant paw edema model

Mouse Alox15 oxygenates AA mainly to 12-HETE [2, 22] but the human ortholog (ALOX15) mainly converts this polyenoic fatty acid to 15-HETE [21, 40]. Since 15-lipoxygenating ALOX15 orthologs may more effectively convert ALOX5-derived precursors to specialized pro-resolving mediators (SPMs) than 12-lipoxygenating enzymes [28, 31], it was expected that *Alox15-KI* mice might develop less intense inflammatory symptoms in mouse inflammation models. To test this hypothesis, we compared the degree of inflammation of *Alox15-KI* mice and of wildtype control animals in two independent mouse inflammation models. In the DSS colitis model we found that *Alox15-KI* mice were protected from the development of inflammatory symptoms as indicated by the differential body weight kinetics (Fig. 2A), by the degree of colon shortening (Fig. 2B), by quantification of histological inflammation markers (Fig. 2C) and by the expression profiles of pro-inflammatory gene products (Fig. 2D–F). In contrast, in the Freund’s complete adjuvant induced paw edema model we did not see significant differences between the two genotypes. In fact, when we quantified the degree of paw edema formation (Fig. 7A–C), the expression profiles of classical pro-inflammatory genes (Fig. 7D–G) and the pressure- (Fig. 7H–J) as well as the heat-induced (Fig. 7K–M) pain sensitivity we did not observe significant differences between *Alox15-KI* mice and wildtype controls. Thus, the Alox15 pathway may play different roles in various types of inflammatory diseases.

### Humanization of mouse Alox15 is mirrored on the level of the colon oxylipins

To explore whether the subtle genetic manipulation of the *Alox15* gene (single nucleotide exchange) is mirrored on the level of the tissue lipid we quantified the oxylipins in the colon tissue of *Alox15-KI* mice and of wildtype controls during the time course of the inflammatory reaction. Analyzing the oxygenated EPA and DHA derivates of untreated mice (no DSS application), we observed that in the colon tissue of *Alox15-KI* mice the 12-HEPE (Fig. 4F) and the 14-HDHA (Fig. 5F) levels were lower than in wildtype control animals. This data suggests that at least a share of these metabolites may have been formed via the Alox15 pathway. If this conclusion is correct, one would expect higher colon concentrations of 15-HEPE and 17-HDHA when *Alox15-KI* mice were compared with wildtype controls. This prediction could be confirmed experimentally (Figs. 4D, 5D). Interestingly, for neither of the other HEPE- and HDHA-isomers (internal negative controls), significant differences between the two genotypes were observed when the colon tissue of untreated animals was analyzed.

For the oxygenated AA metabolites, the situation was somewhat different. Here we also observed significantly higher 12-HETE levels in the colon of untreated wildtype animals when compared with untreated *Alox15-KI* mice (Fig. 3D) and these data suggested that wildtype Alox15 may have contributed to the formation of 12-HETE. However, although the 15-HETE concentrations in the colon tissue were somewhat higher in the *Alox15-KI* mice (Fig. 3B), the difference to wildtype controls was statistically not significant. The most plausible explanation for this observation is that the 15-HETE formed by the humanized Alox15 is further converted to secondary products, which were not picked-up by our analytical system.

Linoleic acid (LA) and alpha-linolenic acid (ALA) are converted by mouse Alox15 and its humanized Leu353Phe mutant mainly to 13-HODE and thus, with these fatty acids there is no difference in the reaction specificity of the two enzyme species. In other words, the patterns of the LA- and ALA derived oxylipins in the colon tissue (Fig. 6) does neither provide valuable information about the in vivo activity of wildtype Alox15 nor its humanized mutant version.

### The pro-inflammatory LTB4 and the pro-resolving mediators NPD-1, Mar-2, and RvD5 show similar kinetics during the time course of DSS colitis

Neuroprotection-1 (NPD-1), resolvin D5 (RvD5), and maresin-2 (Mar-2) are specialized pro-resolving mediators, which exhibit anti-inflammatory and pro-resolving effects in different inflammation models [36, 41]. However, the biological role of these lipid mediators have recently been challenged [42] and there is an ongoing debate on the technical details of SPM analysis [43]. Systemic administration of low concentrations of Mar-2 encapsulated in thermostable nanoparticles protected mice from DSS-induced colitis [44] and this data supports the pro-resolving activity of Mar-2 during DSS-induced experimental colitis. In our study we confirmed that Mar-2 is formed in the DSS-induced intestinal inflammation model but when we followed the kinetic of the pro-resolving metabolites NPD-1, Mar-2 and RvD5 during the time course of the disease (Fig. 6H–J) we found low colon levels in untreated mice of either genotype. It was found that 8 days of DSS-treatment induced a strong increase in the metabolite concentrations which returned to normal during the recovery period. A similar time course was observed for the classical pro-inflammatory mediator leukotriene B4 (Fig. 6G) and these similarities are somewhat surprising. The most plausible explanation for these findings is that the inflammatory response induced by DSS administration might instantaneously be followed by a resolving counterresponse and thus, pro-inflammatory and pro-resolving mediators might be synthesized simultaneously.

In wildtype mice we only detected small amounts of RvD5 in normal and inflamed colon tissue (Fig. 6J). In contrast much higher RvD5 levels were detected in the colon of humanized *Alox15-KI* mice. RvD5 (7S,17S-dihydroxy-4Z,8E,10Z,13Z,15E,19Z-docosahexaenoic acid) is a double oxygenation product of DHA and its biosynthesis is likely to involve two consecutive steps of DHA lipoxygenation via the Alox5 (formation of 7S-HDHA) and the Alox15 pathways (formation of 7S,17S-diHDHA). Since wildtype mouse Alox15 has a much lower biosynthetic capacity for the DHA 17-oxygenation than its humanized Leu353Phe mutant, the elevated biosynthetic capacity of *Alox15-KI* mice for RvD5 is plausible. Interestingly, the RvD5 colon concentrations in *Alox15-KI* mice are significantly higher at all time points of intestinal inflammation than in wildtype control animals (Fig. 6J). These results provide a plausible explanation for the observation that *Alox15-KI* mice are more resistant to DSS-induced colitis (Fig. 2A–F).

Comparing the tissue concentrations of the pro-resolving mediators NPD-1 and Mar-2 (Fig. 6H + I) we found that with a single exception (NPD-1, 8 days DSS, Fig. 6H) the metabolite levels were higher in wildtype mice than in *Alox15-KI* animals. For the biosynthesis of Mar-2 (Fig. 6I) this finding can easily be explained. Mar-2 biosynthesis involves the formation of 14-hydroperoxy-DHA, which is formed as major oxygenation product from DHA by wildtype mouse Alox15 [4]. In contrast, the humanized mouse Alox15 exhibits a strongly reduced capacity for the formation of 14-hydroperoxy-DHA since 17-hydro(pero)xy-DHA is the dominant DHA oxygenation product. Thus, Mar-2 biosynthesis in *Alox15-KI* mice was expected to be compromised. Indeed, we observed lower colon tissue concentrations of Mar-2 in *Alox15-KI* mice at all time points of the experimental colitis (Fig. 6I).

NPD-1 biosynthesis involves the intermediate formation of 17S-hydroperoxy-DHA, but this compound is only a minor side product of DHA oxygenation by wildtype mouse Alox15. Humanization of mouse Alox15 augments the relative share of 17-hydroperoxy DHA formation and thus, NPD-1 formation by *Alox15-KI* mice should be elevated. However, we observed significantly lower NPD-1 concentrations in the uninflamed (no DSS) colon tissue of *Alox15-KI* mice but also after the recovery period (Fig. 6H). A possible explanation for this obvious discrepancy is that an augmented biosynthetic capacity of a key enzyme (Alox15 in our case) does not necessarily mean that the tissue concentrations of a given mediator may also be elevated. Tissue steady-state concentrations do not only depend on the efficiency of biosynthesis (input-control) but also on the activity of degrading enzymes (output-control). Unfortunately, little is known on the regulation of NPD-1 degrading reactions during the time-course of experimental colitis and we did not quantify these pathways in the present study.

### Advancement of science and limitations of the study

As most mammalian ALOX15 orthologs [28] mouse Alox15 exhibits an arachidonate 12-lipoxygenating activity and thus, the pattern of polyenoic fatty acid oxygenation products is fundamentally different when mouse and human ALOX15 orthologs are compared. In other words, when it comes to the ALOX15 pathway experimental data obtained in mouse models of human diseases cannot directly be transferred to the human situation. The *Alox15-KI* mice explored here express an Alox15 mutant with humanized reaction specificity making the animals a more suitable model for exploring the role of this enzyme in human diseases. We are currently exploring the impact of the Leu353Phe exchange in the *Alox15* gene in a mouse atherosclerosis model but it would also be worth to test these mice in different mouse cancer models [45] and/or in mouse models of human neurodegeneration [46].

When we started this project, we expected that *Alox15-KI* mice were protected from inflammation since AA 12-lipoxygenating ALOX15 orthologs exhibited a reduced in vitro capacity [31] for pro-resolving lipoxins. Unfortunately, we were not able to detect lipoxin isomers in colon tissue neither during acute inflammation nor in the resolution phase. On the other hand, we detected three different di-hydroxylated SPMs (NPD-1, Mar-2, RvD5) in the colon tissue (Fig. 6H, I, J) and the local RvD5 kinetics during the time-course of intestinal inflammation were consistent with the observed anti-inflammatory effect of our genetic manipulation. Next, we explored the possibility whether functional humanization of mouse Alox15 might modify the pro-inflammatory Alox5 pathway. Although we observed a slightly reduced Alox5 expression in the colon tissue of *Alox15-KI* mice before systemic DSS administration, such genotype-specific differences were not observed at day 8 and day 16 (see Figure S2, additional file 1). Moreover, previous ex vivo activity assays suggested that human blood cells prepared from *Alox15*-*KI* mice and wildtype controls produce similar amounts of LTB4 when stimulated with calcium ionophore [9]. In addition, when we compared the colonic LTB4 levels during the time course of inflammation (Fig. 6G) in *Alox15-KI* mice and in wildtype controls we neither observed significant differences between the two genotypes. Taken together, our subtle genetic manipulation of the *Alox15* gene did not significantly impact the Alox5 pathway and thus, reduction of the formation of pro-inflammatory leukotrienes may not be discussed as major reason for the protective effect of functional humanization of mouse Alox15 in the DSS colitis model. Thus, the molecular basis for the protective effect of Alox15 humanization remains unclear and additional animal experiments are needed to shed light on this topic. In vivo experiments have recently been shown that functional inactivation of the *Alox15* gene improved the colonic barrier function so that *Alox15*^*−/−*^ mice were protected in the DSS colitis model [47]. Functional humanization of the Alox15 pathway might also contribute to the improvement of the enteral barrier function, but for the time being experimental confirmation of this hypothesis is still pending. Corresponding experiments are rather complex and require separate permission and thus, they would exceed the frame of the present study.

Another limitation of our study is the lack of chirality data for the monohydroxylated PUFAs detected in normal and inflamed colon tissue. Such data would have been helpful to judge the relative share of Alox-derived PUFA oxygenation products in relation to other metabolic pathways such as PUFA autoxidation and/or radical mediated lipid peroxidation. However, chiral chromatography has its limitations and interpretation of chirality data is not always straightforward: (1) The amounts of analytes required for proper enantiomer separation are rather high and thus, chiral analysis of tissue lipids requires large amounts of biological samples. In our case the amounts of OH-PUFAs extracted from the colon lipids were not sufficient for proper chiral chromatography. (2) When a mixture of enzymatic and nonenzymatic oxygenation products is expected functional interpretation of the enantiomer composition is problematic. These problems are particularly severe when the ALOX pathway contributes only a minor share to the sum of the oxygenation products. According to the data shown in Figs. 3, 4 and 5 this is the case for the OH-PUFAs present in normal and inflamed colon lipids. (3) A racemic OH-PUFAs pattern (similar amounts of R- and S-enantiomers) suggests nonenzymatic oxidation reactions. However, preponderance of the S- or the R-isomer of a given OH-PUFA does not prove the involvement of an Alox-isoform in oxylipin biosynthesis. For instance, a chiral pattern of OH-PUFA, in which the S-enantiomer dominates, may also be the consequence of an enantio-selective removal of the R-isomer from a racemic mixture formed via nonenzymatic oxygenation processes.

## Conclusion

Functional humanization of mouse Alox15 protected *Alox15* knock-in mice from the development of inflammatory symptoms in the dextran sulfate sodium induced colitis model. This protective effect might be related to the elevated tissue concentrations of resolvin D5 but not to the reduced levels of maresin-1. In the Freund’s complete adjuvant induced paw edema model functional humanization of Alox15 was not protective.

## Supplementary Information

Additional file 1. Additional methodological information and supplemental experimental data which include Figure S1, Figure S2, Table S1, Table S2 and Table S3

## Data Availability

All experimental raw data and all materials used in this study are available from the authors upon request.

## Abbreviations

ALOX15: Arachidonic acid 15-lipoxygenase
PUFA: Polyenoic fatty acids
LA: Linoleic acid
AA: Arachidonic acid
EPA: Eicosapentaenoic acid
DHA: Docosahexaenoic acid
DSS: Dextran sodium sulfate
FCA: Freunds’ complete adjuvant
5-H(p)ETE: 15-Hydro(pero)xy-5*Z*,8*Z*,11*Z*,13*E*-eicosatetraenoic acid
12-H(p)ETE: 12-Hydro(pero)xy-5*Z*,8*Z*,10*E*,14*Z*-eicosatetraenoic acid
11-H(p)ETE: 11-Hydro(pero)xy-5*Z*,8*Z*,12*E*,14*Z*-eicosatetraenoic acid
9-H(p)ETE: 9-Hydro(pero)xy-5*Z*,7*E*,11*Z*,14*Z*-eicosatetraenoic acid
8-H(p)ETE: 8-Hydro(pero)xy-5*Z*,9*E*,11*Z*,14*Z*-eicosatetraenoic acid
5-H(p)ETE: 5S-hydro(pero)xy-6*E*,8*Z*,11*Z*,14*Z*-eicosatetraenoic acid
18-HEPE: 18-Hydroxy-5*Z*,8*Z*,11*Z*,14*Z*,16*E*-eicosapentaenoic acid
15-HEPE: 15-Hydroxy-5*Z*,8*Z*,11*Z*,13*E*,17*Z*-eicosapentaenoic acid
14-HEPE: 14-Hydroxy-5*Z*,8*Z*,11*Z*,13*E*,17Z-eicosapentaenoic acid
12-HEPE: 12-Hydroxy-5*Z*,8*Z*,11*Z*,15*E*,17*Z*-eicosapentaenoic acid
11-HEPE: 11-Hydroxy-5*Z*,8*Z*,12*E*,14*Z*,17*Z*-eicosapentaenoic acid
9-HEPE: 9-Hydroxy-5*Z*,7*E*,11*Z*,14*Z*,17*Z*-eicosapentaenoic acid
8-HEPE: 8-Hydroxy-5*Z*,9*E*,11*Z*,14*Z*,17*Z*-eicosapentaenoic acid
5-HEPE: 5-Hydroxy-6*E*,8*Z*,11*Z*,14*Z*,17*Z*-eicosapentaenoic acid
20-HDHA: 20-Hydroxy-4*Z*,7*Z*,10*Z*,13*Z*,16*Z*,18*E*-docosahexaenoic acid
17-HDHA: 17-Hydroxy-4*Z*,7*Z*,10*Z*,13*Z*,15*E*,19*Z*-docosahexaenoic acid
16-HDHA: 16-Hydroxy-4*Z*,7*Z*,10*Z*,13*Z*,17*E*,19*Z*-docosahexaenoic acid
14-HDHA: 14-Hydroxy-4*Z*,7*Z*,10*Z*,12*E*,16*Z*,19*Z*-docosahexaenoic acid
13-HDHA: 13-Hydroxy-4*Z*,7*Z*,10*Z*,14*E*,16*Z*,19*Z*-docosahexaenoic acid
11-HDHA: 11-Hydroxy-4*Z*,7*Z*,9*E*,13*Z*,16*Z*,19*Z*-docosahexaenoic acid
10-HDHA: 10-Hydroxy-4*Z*,7*Z*,11*E*,13*Z*,16*Z*,19*Z*-docosahexaenoic acid
8-HDHA: 8-Hydroxy-4*Z*,6*E*,10*Z*,13*Z*,16*Z*,19*Z*-docosahexaenoic acid
7-HDHA: 7-Hydroxy-4*Z*,8*E*,10*Z*,13*Z*,16*Z*,19*Z*-docosahexaenoic acid
4-HDHA: 4-Hydroxy-5*E*,7*Z*,10*Z*,13*Z*,16*Z*,19*Z*-docosahexaenoic acid
LTB4: Leukotriene B4 (5*S*,12*R*-dihydroxy-6*Z*,8*E*,10*E*,14*Z*-eicosatetraensäure
NPD1: Neuroprotection-1 (10R,17*S*-dihydroxy-4*Z*,7*Z*,11*E*,13*E*,15*Z*,17*Z*-docosahexaenoic acid)
RvD5: Resolvin-D5 (7*S*,17*S*-dihydroxy-4*Z*,8*E*,10*Z*,13*Z*,15*E*,19*Z*-docosahexaenoic acid)
13-HODE: 13-Hydroxy-9*Z*,11*E*-octadecadienoic acid
9-HODE: 9-Hydroxy-10*E*,12*Z*-octadecadienoic acid
13-HOTrE: 13-Hydroxy-9*Z*,11*E*,15*Z*-octadecatrienoic acid
9-HOTrE: 9-Hydroxy-10*E*,12*Z*,15*Z*-octadecatrienoic acid
