# Supplementary material for: Functional humanization of 15-lipoxygenase-1 (Alox15) protects mice from dextran sodium sulfate induced intestinal inflammation
Source: Cell Mol Biol Lett. 2025 Jul 13;30:81. doi: 10.1186/s11658-025-00756-0 (PMC12255980; doi:10.1186/s11658-025-00756-0)
Supplement: Supplementary file 1 — Additional file 1. Additional methodological information and supplemental experimental data which include Figure S1, Figure S2, Table S1, Table S2 and Table S3 [file 11658_2025_756_MOESM1_ESM.pdf]

Supplemental data for the paper

Functional humanization of 15-lipoxygenase protects mice from dextran sodium sulfate induced intestinal inflammation

Florian Reisch[1,2], Marjann Schäfer[1,2], Dominika Labuz[3], Halina Machelska[3], Sabine Stehling[1], Gerhard P. Püschel[2], Michael Rothe[4], Dagmar Heydeck[1], and Hartmut Kuhn[1]*

[1]Department of Biochemistry, Charité – Universitätsmedizin Berlin, corporate member of Freie Universität Berlin and Humboldt Universität zu Berlin, Charitéplatz 1, D-10117 Berlin, Germany.

[2]Institute for Nutritional Sciences, University Potsdam, Arthur-Scheunert-Allee 114-116, D-14558 Nuthetal, Germany

[3]Department of Experimental Anesthesiology, Charité - Universitätsmedizin Berlin, corporate member of Freie Universität Berlin and Humboldt-Universität zu Berlin, Hindenburgdamm 30, D-12203 Berlin;

[4]Lipidomix GmbH, Robert-Roessle-Str. 10, 1D-3125 Berlin, Germany

Running title: Alox15 KI mice in inflammation models

Keywords: eicosanoids, lipid peroxidation, oxylipidomes, inflammation, pain, colitis, paw edema

***Address: correspondence to:** hartmut.kuehn@charite.de

1. Supplemental experimental data

Figure S1: LC-MS/MS analyses of selected specialized pro-resolving mediators. Colitis induction, sample workup and LC-MS/MS analysis were carried out as described in Materials and Methods. The retention times of authentic standards are indicated by the arrows above the chromatographic traces. For RvD1, LxA4, RvD2, RvD3, LxB4 and RvD4 no clear-cut chromatographic peaks were observed. In contrast, for RvD5, NPD1and Mar2 quantifiable peaks were detected.

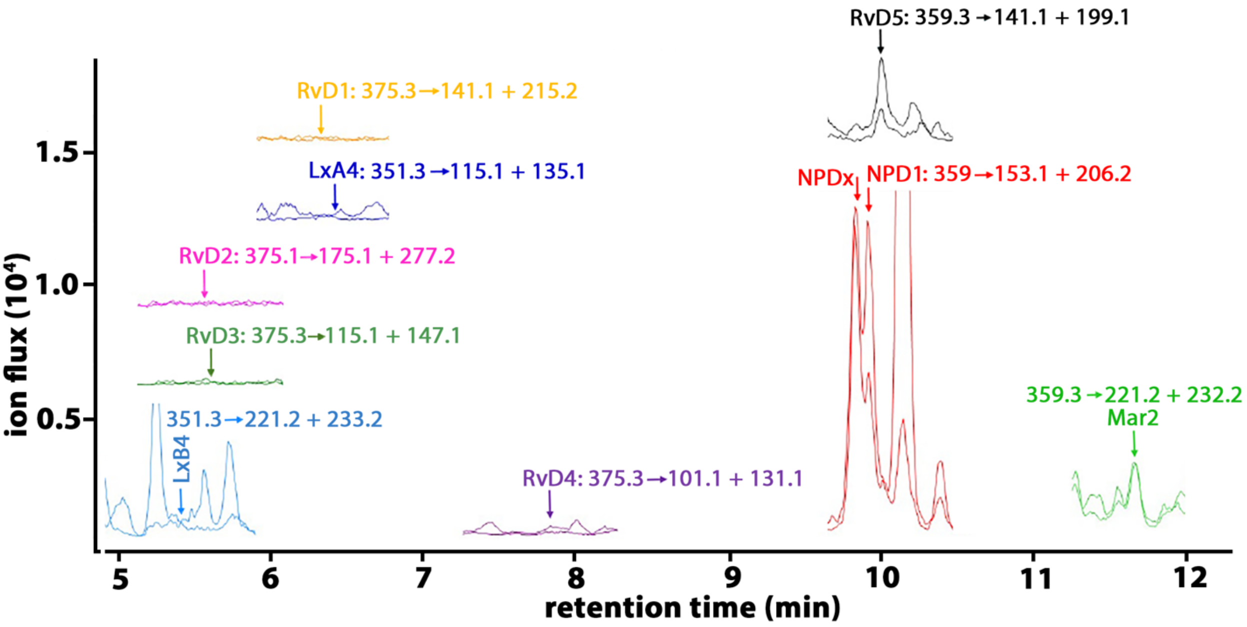

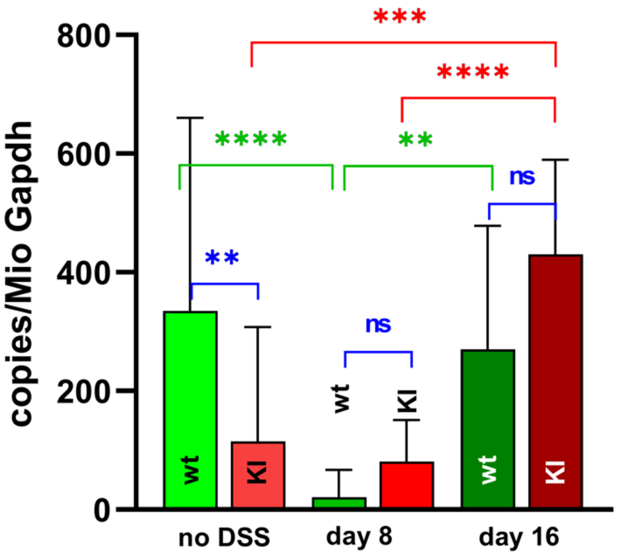

Figure S2: Quantitative RT-PCR of the Alox5 mRNA in colon tissue at different time points of experimental colitis. 10-15 mg (wet weight) of inflamed colon tissue were prepared at different time points of the inflammatory reaction and stored in RNAlater solution (Sigma-Aldrich/Merck, Taufkirchen, Germany) at -20° C. After thawing the tissue was cut into small pieces and was then homogenized in 400 µl LBP buffer (Nucleospin RNA plus kit, Macherey-Nagel, Düren, Germany) using a FastPrep24 homogenizer. Cell debris was spun down and from the homogenate supernatant total RNA was extracted. Subsequently, 500 ng RNA were reversely transcribed using the Tetro Reverse Transcriptase kit (Meridian Bioscience, Memphis, USA, distributed by BioCat GmbH, Heidelberg, Germany) and Oligo dT_{18} reagents as recommended by the vendor. qRT-PCR of Alox5 mRNA was performed using the following amplification primer: forward, 5'-TCG AGT TCC CAT GTT ACC GCT-3' and reverse 3'-CTG TGG TCA CTG GGA GCT TCG-5'. The concentration of the target cDNA was quantified using standard curves (known copy numbers of the external amplification standards) and the amounts of amplification products were normalized to Gapdh expression. qRT-PCR was performed on a Rotor Gene 3000 device (Corbett Research, Mortlake, Australia). The progress of the amplification process was followed online using the SensiMix^{TM} SYBR PCR Kit (Meridian Bioscience, Memphis, TN, USA, distributed by BioCat GmbH, Heidelberg, Germany). The experimental raw data were evaluated statistically with the two-way ANOVA function of the GraphPad Prism program.

Table S1: Detection limits for the hydroxy fatty acids analyzed in this study.

No.	Parent fatty acid	Metabolites	Detection limit (ng)
1	Docosahexaenoic acid	4-HDHA	0.13
2		7-HDHA	0.22
3		8-HDHA	0.43
4		8-HDHA	0.43
5		11-HDHA	0.14
6		13-HDHA	0.62
7		14-HDHA	0.11
8		16-HDHA	0.12
9		17-HDHA	0.38
10		20-HDHA	0.18
11	Eicosapentaenoic acid	5-HEPE	0.16
12		8-HEPE	0.17
13		9-HEPE	0.36
14		11-HEPE	2.11
15		12-HEPE	0.18
16		15-HEPE	0.29
17		18-HEPE	0.21
18	Arachidonic acid	5-HETE	0.16
19		8-HETE	1.97
20		9-HETE	1.88
21		11-HETE	0.20
22		12-HETE	0.27
23		15-HETE	0.20
24	Linoleic acid	9-HODE	0.24
25		13-HODE	0.12
26	Linolenic acid	9-HOTrE	0.34
27		13-HOTrE	0.34
28	Dihomo linolenic acid	8-HeTrE	0.37
29		12-HETrE	0.20
30		15-HeTrE	0.20

Table S2: Detection limits for the complex oxylipins analyzed in this study.

No.	Metabolites	Detection limit (ng)
1	NPD-1	0.14
2	Maresin-1	0.63
3	Maresin-2	0.12
4	RvD1	0.43
5	RvD1 17(R)	0.56
6	RvD2	0.63
7	RvD3	0.55
8	RvD4 17(R,S)	0.41
9	RvD5	0.28
10	LTB4	0.11
11	LTB4 18-COOH dinor	0.37
12	LTB3	0.12
13	PGJ2 15-deoxy-delta 12,14	n.d.
14	PGB-3	n.d.
15	PGB-2	n.d.
16	LTB4 12-oxo	0.10
17	LTB5 (EPA)	0.12
18	PGJ2	n.d.
19	PGJ2 delta12	n.d.
20	LXA5 (EPA)	0.15
21	LXA4 15(R)epi	0.11
22	LXA4 6(S)	0.12
23	LXA4	0.26
24	LXB4	0.13

Table S3: Scoring of inflammatory symptoms. We evaluated the histological preparations of colon tissue for inflammatory symptoms and quantified three morphological inflammation parameters according to a semi-quantitative scoring system (see ref. 33): i) extent of neutrophil infiltration, ii) extent of epithelial defects, iii) extent of mucosal ulcerations. Score 0: No signs of inflammation. 1: low signs of inflammation. 2: Medium signs of inflammation. 3: Strong signs of inflammation.

samples	Score ± SD	Description
Wildtype, day 0, no DSS	0.00 ± 0.00	no signs of inflammation
Alox15-KI, day 0, no DSS	0,00 ± 0.00	no signs of inflammation
Wildtype, day 8, with DSS	1,33 ± 0.82	diffuse mild inflammation of mucosa and submucosa
Alox15-KI, day 8, with DSS	1,17 ±0.41	minimal to mild multifocal inflammation of mucosa
Wildtype, day 16, with DSS	0,80 ± 0.00	minimal to mild multifocal inflammation of mucosa
Alox15-KI, day 16, with DSS	1,00 ± 0.45	Mild multifocal inflammation of mucosa

2. Methodological supplemental information

2.1. HPLC analysis of the ALOX products formed during in vitro and ex-vivo activity assays

RP-HPLC was carried out using a Shimadzu instrument (LC20 AD) that was equipped with a diode array detector (SPD M20A). The hydroxy fatty acids were separated on a Nucleodur C18 Gravity column (Macherey-Nagel, Düren, Germany; 250 x 4 mm, 5 µm particle size) coupled with a guard column (8 x 4 mm, 5 µm particle size). A solvent system consisting of acetonitrile : water : acetic acid (70:30:0.1, by vol) was employed at a flow rate of 1 ml/min and analytes were eluted isocratically at 25° C. The conjugated dienes formed during the incubation period were prepared, the solvents were removed and the remaining lipids were reconstituted in 200 µL of hexane containing 0.1 % acetic acid.

To resolve the hydroxy fatty acid isomers formed during the incubation period combined normal phase/chiral phase HPLC (NP/CP-HPLC) was carried out. For this purpose, a Chiralpak AD-H column (4.6 x 250 mm, 5 µm particle size, Daicel (Osaka, Japan) was connected with a Nucleosil pre-column (4.6 x 30 mm, 5 µm particle size, Macherey-Nagel (Düren, Germany) and the analytes were eluted isocratically using a solvent system consisting of n-hexane / methanol / ethanol / acetic acid (96 / 3 / 1 / 0.1, by vol) at a flow rate of 1 ml/min. The absorbance at 235 nm was monitored and the retention times of authentic standards are indicated above the chromatographic traces.

2.2. LC-MS/MS based oxylipidome analyses

To explore the patterns of the colon oxylipins we quantified the amounts of more than 40 different oxygenated PUFA derivatives **(23, 24)**. LC-MS/MS was performed on an Agilent 1290/II LC-MS system consisting of a binary pump, an autosampler and a column oven (Agilent Technologies, Waldbronn, Germany). For chromatographic separation, we employed an Agilent Zorbax Eclipse C_{18} UPLC column (150 x 2.1 mm, 1.8 µm particle size). The analytes were eluted at 30° C with a solvent system that was mixed from two solvent stock solutions. Stock A: Water containing 0.05 % acetic acid. Stock B: 1:1 mixture (by vol.) of methanol : acetonitrile. The HPLC system was connected with a triple quadrupole MS system (Agilent 6495 System, Agilent Technologies, Santa Clara, USA) that was run in the negative electrospray ionization mode. Each metabolite was detected simultaneously by two independent mass transitions. Experimental raw data were evaluated with the Agilent Mass-Hunter software package, version B10.0. For all metabolites analyzed in this study individual calibration curves were set up and the lower detection limits were determined **(Table S1+S2)**.
